# Risk Factors for Severe Disease Among Children Hospitalized With Respiratory Syncytial Virus

**DOI:** 10.1001/jamanetworkopen.2025.4666

**Published:** 2025-04-11

**Authors:** Nardin Kirolos, Haifa Mtaweh, Rohini R. Datta, Daniel S. Farrar, Claire Seaton, Jeffrey N. Bone, Fiona Muttalib, Caitlyn L. Kaziev, Jonathan Fortini, Sanjay Mahant, Aaron Campigotto, Gabrielle Freire, Rae S. M. Yeung, Jonathan H. Rayment, Connie Yang, Jocelyn A. Srigley, Manish Sadarangani, Francine Buchanan, Shaun K. Morris, Peter J. Gill

**Affiliations:** 1 Department of Pediatrics, University of Toronto, Toronto, Ontario, Canada; 2 Interdepartmental Division of Critical Care Medicine, The Hospital for Sick Children, Toronto, Ontario, Canada; 3 Child Health Evaluative Sciences, SickKids Research Institute, Toronto, Ontario, Canada; 4 Centre for Global Child Health, The Hospital for Sick Children, Toronto, Ontario, Canada; 5 Division of Pediatric Hospital Medicine, BC Children’s Hospital, Vancouver, British Columbia, Canada; 6 Department of Pediatrics, University of British Columbia, Vancouver, British Columbia, Canada; 7 BC Children’s Hospital Research Institute, Vancouver, British Columbia, Canada; 8 Biostatistics Clinical Research Support Unit, BC Children’s Hospital Research Institute, Vancouver, British Columbia, Canada; 9 Division of Pediatric Critical Care Medicine, BC Children’s Hospital, Vancouver, British Columbia, Canada; 10 Division of Paediatric Medicine, The Hospital for Sick Children, Toronto, Ontario, Canada.; 11 Institute of Health Policy, Management, and Evaluation, University of Toronto, Toronto, Ontario, Canada; 12 Division of Microbiology, The Hospital for Sick Children, Toronto, Ontario, Canada; 13 Department of Laboratory Medicine and Pathobiology, University of Toronto, Toronto, Ontario, Canada; 14 Division of Paediatric Emergency Medicine, The Hospital for Sick Children, Toronto, Ontario, Canada; 15 Division of Rheumatology, The Hospital for Sick Children, Toronto, Ontario, Canada; 16 Cell Biology, SickKids Research Institute, The Hospital for Sick Children, Toronto, Ontario, Canada; 17 Department of Immunology, University of Toronto, Toronto, Ontario, Canada; 18 Department of Medical Sciences, University of Toronto, Toronto, Ontario, Canada; 19 Division of Respiratory Medicine, BC Children’s Hospital, Vancouver, British Columbia, Canada; 20 Division of Paediatric Infectious Disease, BC Children’s Hospital, Vancouver, British Columbia, Canada; 21 Department of Pathology and Laboratory Medicine, University of British Columbia, Vancouver, British Columbia, Canada; 22 Vaccine Evaluation Center, BC Children’s Hospital, Vancouver, British Columbia, Canada; 23 Centre for Innovation and Excellence in Child and Family-Centred Care, The Hospital for Sick Children, Toronto, Ontario, Canada; 24 Division of Infectious Diseases, The Hospital for Sick Children, Toronto, Ontario, Canada; 25 Dalla Lana School of Public Health, University of Toronto, Toronto, Ontario, Canada; 26 Temerty Faculty of Medicine, University of Toronto, Toronto, Ontario, Canada

## Abstract

**Importance:**

A resurgence of respiratory syncytial virus (RSV)–associated acute respiratory tract infection (ARI) was observed in 2022 and 2023 after the COVID-19 pandemic. Changes in the demographic characteristics, disease severity, and outcomes of patients were observed, which could impact the identification of risk groups for interventions aimed at reducing the severity of RSV disease.

**Objectives:**

To identify factors associated with severe clinical outcomes among children hospitalized with RSV-associated ARIs in 2022 and 2023.

**Design, Setting, and Participants:**

This observational cohort study, conducted at 2 large, Canadian, tertiary-level pediatric hospitals, comprised all 709 cases of RSV-associated ARI among children younger than 18 years who were admitted to the hospital or intensive care unit (ICU) from July 1, 2022, to June 30, 2023.

**Exposure:**

Diagnosis of RSV-associated ARI.

**Main Outcomes and Measures:**

The primary outcome of severe disease was defined as requiring noninvasive or invasive ventilation or death. Risk factors for severe disease and ICU admission (secondary outcome) were assessed using multivariable Poisson regression, and results were reported as adjusted risk ratios (ARRs) with 95% CIs, with age-stratified models (<2 years and ≥2 years).

**Results:**

A total of 709 cases (median age, 13.1 months [IQR, 2.0-36.6 months]; 442 boys [62.3%]) were admitted with RSV-associated ARI; 452 (63.8%) were younger than 2 years, and 257 (36.2%) were aged 2 years or older. Severe disease was documented for 204 cases (28.8%). Patients with severe disease were younger than those with nonsevere disease (median age, 2.6 months [IQR, 1.3-16.0 months] vs 18.6 months [IQR, 4.5-39.1 months]; *P* < .001). Pulmonary disease and use of home oxygen (ARR, 2.47 [95% CI, 1.30-4.68]) and neurologic, neuromuscular, and developmental conditions (ARR, 1.89 [95% CI, 1.03-3.49]) were associated with severe disease among children aged 2 years or older. Among children younger than 2 years, age younger than 3 months (ARR, 2.34 [95% CI, 1.43-3.84]), age 3 to less than 6 months (ARR, 2.79 [95% CI, 1.65-4.70]), and prematurity (ARR, 1.40 [95% CI, 1.03-1.89]) were associated with severe disease.

**Conclusions and Relevance:**

In this cohort study of children hospitalized with RSV in 2022 and 2023, severe RSV disease was more likely among those aged 2 years or older with pulmonary and neurologic, neuromuscular, or developmental conditions. For children younger than 2 years, age younger than 6 months and prematurity were the main risk factors. These findings support prevention strategies for all younger children, including premature infants, with potential benefit for children aged 2 years or older in specific high-risk groups.

## Key Points

**Question:**

What are the current risk factors for severe respiratory syncytial virus (RSV) infection given the changing epidemiologic characteristics after the COVID-19 pandemic?

**Findings:**

In this cohort study that included 709 cases of RSV-associated acute respiratory tract infection, severe disease was more likely among children aged 2 years or older with pulmonary and neurologic, neuromuscular, or developmental conditions; children younger than 2 years with a history of prematurity; or children younger than 6 months.

**Meaning:**

Given the identified risk groups, this study suggests there may be benefit associated with prevention strategies, such as universal prophylaxis with monoclonal antibodies in infancy and for children aged 2 years or older in specific high-risk groups.

## Teaser

This cohort study assesses risk factors associated with severe clinical outcomes among children hospitalized with respiratory syncytial virus–associated acute respiratory tract infections in 2022 and 2023.

## Introduction

Respiratory syncytial virus (RSV) is the primary cause of acute lower respiratory tract infections among infants and young children, leading to high rates of emergency department visits, hospitalizations, and intensive care unit (ICU) admissions, with a major impact on families and health care systems.^1^ In 2019, RSV infections caused an estimated 3.6 million hospital admissions and more than 100 000 deaths among children younger than 5 years globally.^1^ During the COVID-19 pandemic, public health measures designed to reduce SARS-CoV-2 transmission contributed to a marked decrease in RSV transmission.^2^ However, the ease of these measures was associated with an out-of-season resurgence in RSV infections, overwhelming pediatric health care facilities with extremely high volumes of acute respiratory tract infections (ARIs).^3,4,5,6^

Studies prior to and during the COVID-19 pandemic identified independent risk factors associated with severe RSV infection, including young age, prematurity, and the presence of comorbidities—in particular, cardiovascular, pulmonary, and certain genetic conditions (eg, trisomy 21).^7,8^ During the 2022-2023 respiratory viral season, there was an increase in RSV-associated ARIs along with changes in patient characteristics and clinical presentations, including older age, longer hospital stays, and increased numbers of ICU admissions.^3,5,6,9^ These studies suggest possible changes in sociodemographic and clinical risk factors associated with severe disease, warranting further investigation. Current literature focuses predominantly on risk factors among younger children, with less known about older children presenting with RSV-associated ARI.

For more than 2 decades, palivizumab has been the primary intervention for RSV prophylaxis; however, its high cost and monthly injection requirement limited its use to infants at high risk.^10,11^ Nirsevimab was introduced as the first long-acting monoclonal antibody among a group of several other candidates; it offers extended protection throughout the RSV season and is now the first immunization product for universal RSV prophylaxis among infants.^12,13^ Internationally, health agencies such as the National Advisory Committee on Immunization,^14^ European Medicines Agency,^15^ Advisory Committee on Immunization Practices,^16^ and Spanish National Immunization Technical Advisory Group^17^ all have broad recommendations for universal use of nirsevimab for infants and children up to 2 years old at high risk. Recent literature from Spain shows that implementation of nirsevimab for all infants younger than 6 months and those aged 6 to 24 months with high-risk conditions was associated with an 82% reduction in RSV-related hospitalizations.^18^ Similarly, in Luxembourg, nirsevimab resulted in a 69% reduction in RSV-related lower respiratory tract infections for infants younger than 6 months.^19^

In this study, we describe sociodemographic characteristics and outcomes of children admitted to 2 large, Canadian, pediatric tertiary care hospitals with RSV-associated ARI from July 2022 to June 2023. Our primary goal was to identify risk factors for severe disease among hospitalized children with RSV-associated ARI, focusing on traditionally affected younger children as well as older children, whose incidence of RSV-associated ARI has increased in the post–COVID-19 era and has not yet been extensively studied. By understanding these potential changes in epidemiologic characteristics and risk factors, we aim to inform evidence-based strategies for preventing severe RSV disease.

## Methods

### Study Design

We conducted a retrospective cohort study using data collected for clinical care at The Hospital for Sick Children (SickKids) in Toronto, Ontario, and British Columbia Children’s Hospital in Vancouver, British Columbia, 2 large, tertiary care pediatric hospitals in Canada. Data were extracted from patient medical records into a Research Electronic Data Capture (REDCap) database.^20,21^ This study was approved by the research ethics boards at SickKids and the University of British Columbia with a waiver of consent for secondary use of data. For patients who were recruited prospectively, oral informed consent was provided by parents; child assent to be involved in the study was also obtained where applicable (eg, child was present and able to understand the study and what is involved in participation). This study followed the Strengthening the Reporting of Observational Studies in Epidemiology (STROBE) reporting guideline for observational studies.^22^

### Study Population

This is a substudy of the Clinical Characteristics and Outcomes of Hospitalized Children With Acute Respiratory Infections (READAPT-Kids) study. In the READAPT-Kids study, we identified all cases of children younger than 18 years admitted in 2022 and 2023 with ARIs. Here, we present all cases of children younger than 18 years admitted with microbiologically confirmed RSV-associated ARI, defined as a primary diagnosis of acute respiratory tract illness (ie, bronchiolitis, croup, asthma exacerbation, and pneumonia [viral or bacterial], including complicated pneumonia, acute respiratory distress syndrome, or respiratory failure), from July 1, 2022, to June 30, 2023. Microbiologic diagnosis was established via either polymerase chain reaction or rapid antigen test. At both hospitals, respiratory viral testing was completed at the discretion of the clinical care professional. Patients were initially identified by admission and discharge diagnoses using *International Classification of Diseases, Tenth Revision, Canada* codes derived from a modified version of the Pediatric Clinical Classification System.^23,24^ Next, patient records were manually screened for inclusion by trained research assistants. Children were excluded if their hospital admission was elective or due to trauma, if they were postoperative with a confirmed alternative diagnosis, or if their infection was hospital acquired. Patients with repeat hospital admissions more than 30 days apart were eligible for inclusion as distinct cases.

### Study Definitions and Outcomes

The primary outcome was severe disease, defined as a composite variable of noninvasive ventilation (continuous or bilevel positive airway pressure), invasive mechanical ventilation, or death during hospital admission. Disease severity was analyzed as a binary variable, with each case being categorized as either severe disease or nonsevere disease. We also evaluated admission to the ICU as our main secondary outcome, encompassing admissions to the pediatric ICUs, cardiac critical care units, and neonatal ICUs. Criteria for ICU admission at both institutions included escalating respiratory requirements beyond heated high-flow nasal cannula (HFNC) settings of 2 L/min/kg and Fio_2_ greater than 60%, requirement for noninvasive (ie, continuous positive airway pressure [CPAP] or bilevel positive airway pressure [BiPAP]) and/or invasive ventilation, requirement of vasoactive support, or clinical deterioration requiring ICU-level monitoring and management. Other secondary outcomes included length of hospital admission (days), length of ICU admission (days), noninvasive and invasive ventilation, severe complications (air leak syndrome, need for chest tube insertion, cardiorespiratory arrest, extracorporeal membrane oxygenation, plasmapheresis, or kidney replacement therapy), hospital death, unscheduled return visit to the emergency department within 30 days, and readmission to our 2 hospitals within 30 days. High-flow nasal cannula was not categorized as a form of noninvasive ventilation for several reasons: HFNC is generally used on the general pediatric wards, while CPAP and BiPAP are typically used only in the ICU; elevated HFNC settings greater than 2 L/min/kg and Fio_2_ greater than 60% are uncommonly a sole reason for ICU admission; and there is wide variability with which HFNC is initiated.

We investigated risk factors based on existing literature and RSV guidelines.^7,14,16,17,25,26,27,28,29,30^ These included demographic factors (age and sex), symptoms at presentation, respiratory viral coinfections, comorbidities (complex care program enrollment and conditions including asthma [including use of inhalers without a formal asthma diagnosis]; pulmonary [including baseline home oxygen use, which includes noninvasive ventilation use]; cardiac; kidney; hematologic; gastrointestinal; neurologic, neuromuscular, or developmental; and chromosomal or genetic diseases), and prematurity (gestational age <37 weeks for those <2 years of age).^25,26,28^

### Statistical Analysis

Demographic and clinical characteristics were described; frequencies and proportions were used for categorical variables, and median (IQR) values were used for continuous variables. Patient characteristics were compared by disease severity (severe or nonsevere) and by age category (<3 months, 3-5 months, 6-11 months, 12-23 months, 2-4 years, and 5 to <18 years), with differences assessed using χ^2^ tests and Fisher exact tests for categorical variables and Wilcoxon rank sum tests and Kruskal-Wallis tests for continuous variables as appropriate.

Risk factors for severe disease and ICU admission were identified using multivariable Poisson regression with robust SEs and summarized using crude risk ratios (RRs) and adjusted RRs (ARRs), with the corresponding 95% CI and *P* value.^31^ The primary model was defined a priori and included sex, age in years, comorbidities (any vs none), transfer from another hospital, respiratory tract viral coinfection (any vs none), and duration of symptoms prior to hospital admission (a linear term measured in days). Age (years) was fit with a restricted cubic spline of 3 knots—placed at 3 months, 1 year, and 2 years—to account for the hypothesized nonlinear association between age and risk of severe disease outcome. We also adjusted for hospital site to account for any site differences. To assess the association between each comorbidity subgroup, the “any comorbidity” variable was substituted with each specific comorbidity subgroup in separate models. The same approach was used to assess specific viral coinfections in place of “any viral coinfection.” After this initial multivariable analysis, we repeated the procedure in models stratified by patient age (ie, <2 and ≥2 years separately). We observed that age category modified the association between comorbidities and the severe disease outcome, and we used this as rationale to present age-stratified results as our primary findings. To evaluate the association of hospital transfer with the findings, we performed a subanalysis for the severe disease outcome, which was restricted to only nontransfer patients.

Finally, a median regression analysis was performed to identify factors associated with hospital length of stay. All statistical tests were conducted at the 5% level of significance and used 2-sided hypothesis testing. All analyses were completed using Stata, version 18 (StataCorp LLC).^32^

## Results

### Description of Study Population

There were 709 cases hospitalized with an RSV infection (eFigure 1 in Supplement 1), with a median age of 13.1 months (IQR, 2.0-36.6 months), 442 boys (62.3%), 267 girls (37.7%), and 291 patients (41.0%) transferred from another hospital (Table 1). Four patients were admitted twice within the 2022-2023 season with RSV-associated ARI, more than 30 days apart, and were classified as separate cases for each of their admissions.

**Table 1.** Sociodemographic Characteristics of Children and Youths Hospitalized With Respiratory Syncytial Virus–Confirmed Acute Respiratory Tract Infections by Severity

Characteristic	Total (N = 709)	Disease severity	
		Nonsevere (n = 505)	Severe (n = 204)
Sex, No. (%)			
Female	267 (37.7)	192 (38.0)	75 (36.8)
Male	442 (62.3)	313 (62.0)	129 (63.2)
Age, median (IQR), mo	13.1 (2.0-36.6)	18.6 (4.5-39.1)	2.6 (1.3-16.0)
Age, No. (%)			
<3 mo	224 (31.6)	114 (22.6)	110 (53.9)
3 to <6 mo	53 (7.5)	25 (5.0)	28 (13.7)
6 to <12 mo	66 (9.3)	55 (10.9)	11 (5.4)
12 to <24 mo	109 (15.4)	96 (19.0)	13 (6.4)
2 to <5 y	183 (25.8)	160 (31.7)	23 (11.3)
5 to <18 y	74 (10.4)	55 (10.9)	19 (9.3)
Chronic comorbid conditions, No. (%)^a^	223 (31.5)	177 (35.0)	46 (22.5)
Cardiac disease, including congenital heart disease	63 (8.9)	49 (9.7)	14 (6.9)
Asthma	54 (7.6)	42 (8.3)	12 (5.9)
Genetic disease	57 (8.0)	44 (8.7)	13 (6.4)
Trisomy 21	17 (2.4)	15 (3.0)	2 (1.0)
Gastrointestinal disease	48 (6.8)	37 (7.3)	11 (5.4)
Complex care program	21 (3.0)	15 (3.0)	6 (2.9)
Neurologic disorder	40 (5.6)	32 (6.3)	8 (3.9)
Cerebral palsy	6 (0.8)	4 (0.8)	2 (1.0)
Epilepsy	20 (2.8)	16 (3.2)	4 (2.0)
Pulmonary disease (not asthma)	35 (4.9)	27 (5.3)	8 (3.9)
Cystic fibrosis	0	0	0
Chronic lung disease or bronchopulmonary dysplasia	17 (2.4)	14 (2.8)	3 (1.5)
Developmental disorder	31 (4.4)	23 (4.6)	8 (3.9)
Use of inhalers without a formal asthma diagnosis	27 (3.8)	21 (4.2)	6 (2.9)
Hematologic disease	17 (2.4)	13 (2.6)	4 (2.0)
Chronic kidney disease	17 (2.4)	14 (2.8)	3 (1.5)
Congenital airway abnormalities	12 (1.7)	9 (1.8)	3 (1.5)
Use of oxygen at home	13 (1.8)	6 (1.2)	7 (3.4)
Malignant neoplasm	10 (1.4)	10 (2.0)	0
Immunosuppressive medication at time of admission	8 (1.1)	8 (1.6)	0
Neuromuscular disorders	4 (0.6)	3 (0.6)	1 (0.5)
Inborn error of metabolism	6 (0.8)	5 (1.0)	1 (0.5)
Immunodeficiency	1 (0.1)	0	1 (0.5)
Other	40 (5.6)	31 (6.1)	9 (4.4)
Patients <2 y, No. (%)	452 (63.8)	290 (57.4)	162 (79.4)
Preterm birth, No./total No. of cases <2 y (%)			
24-25 wk	3/452 (0.7)	3/290 (1.0)	0
26-27 wk	4/452 (0.9)	3/290 (1.0)	1/162 (0.6)
28-31 wk	11/452 (2.4)	5/290 (1.7)	6/162 (3.7)
32-34 wk	18/452 (4.0)	9/290 (3.1)	9/162 (5.6)
35-36 wk	40/452 (8.8)	20/290 (6.9)	20/162 (12.3)
Term (≥37 wk)	360/452 (79.6)	235/290 (81.0)	125/162 (77.2)
Unknown	16/452 (3.5)	15/290 (5.2)	1/162 (0.6)
Patients <3 y, No. (%)	525 (74.0)	357 (70.7)	168 (82.4)
Received palivizumab, No./total No. of cases <3 y (%)	16/525 (3.0)	14/357 (3.9)	2/168 (1.2)
Hospital admission pathway			
Direct admission	3 (0.4)	3 (0.6)	0
Emergency department	415 (58.5)	378 (74.9)	37 (18.1)
Hospital transfer	291 (41.0)	124 (24.6)	167 (81.9)

^a^ Patients may have had multiple chronic comorbid conditions, resulting in a sum greater than 100%.

Of the 709 RSV-associated ARI hospitalizations, 204 (28.8%) met criteria for severe disease (Table 1). Children with severe disease were younger than those with nonsevere disease (median age, 2.6 months [IQR, 1.3-16.0 months] vs 18.6 months [IQR, 4.5-39.1 months]; *P* < .001). Almost two-thirds of children (452 [63.8%]) were younger than 2 years, of whom 76 (16.8%) had a history of prematurity. Almost one-third (223 [31.5%]) of cases had at least 1 documented comorbid condition, with the most common diagnoses being neurologic, neuromuscular, and neurodevelopmental conditions (75 [10.6%]); cardiac disease (63 [8.9%]); genetic disease (57 [8.0%]); and asthma (54 [7.6%]).

### Clinical Presentation and Microbiological Investigations

The median duration of symptoms prior to hospital admission was 4 days (IQR, 3-5 days), which was similar between the severe and nonsevere groups (Table 2). The most common symptoms at presentation included cough (629 [88.7%]), increased work of breathing (536 [75.6%]), fever (482 [68.0%]), and nasal congestion (453 [63.9%]). Increased work of breathing, apnea, tachypnea, and cyanosis were more common among children with severe disease vs nonsevere disease. The most common discharge diagnosis was bronchiolitis (436 [61.5%]).

**Table 2.** Clinical Features and Characteristics of Children Hospitalized With Respiratory Syncytial Virus–Confirmed Acute Respiratory Tract Illness by Severity

Characteristic	Total (N = 709)	Disease severity	
		Nonsevere (n = 505)	Severe (n = 204)
Duration of symptoms prior to admission, median (IQR), d	4 (3-5)	4 (3-5)	4 (2-5)
Symptoms at presentation, No. (%)^a^			
Cough	629 (88.7)	460 (91.1)	169 (82.8)
Increased work of breathing	536 (75.6)	358 (70.9)	178 (87.3)
Fever	482 (68.0)	381 (75.4)	101 (49.5)
Nasal congestion	453 (63.9)	317 (62.8)	136 (66.7)
Poor feeding or appetite	343 (48.4)	246 (48.7)	97 (47.5)
Vomiting	200 (28.2)	160 (31.7)	40 (19.6)
Fatigue or lethargy	202 (28.5)	141 (27.9)	61 (29.9)
Dehydration or reduced urine output	127 (17.9)	89 (17.6)	38 (18.6)
Fast breathing or tachypnea	123 (17.3)	72 (14.3)	51 (25.0)
Wheezing	102 (14.4)	78 (15.4)	24 (11.8)
Cyanosis	86 (12.1)	43 (8.5)	43 (21.1)
Shortness of breath	80 (11.3)	45 (8.9)	35 (17.2)
Diarrhea	62 (8.7)	49 (9.7)	13 (6.4)
Irritability	50 (7.1)	31 (6.1)	19 (9.3)
Conjunctivitis	33 (4.7)	24 (4.8)	9 (4.4)
Apnea	33 (4.7)	11 (2.2)	22 (10.8)
Increased secretions	30 (4.2)	15 (3.0)	15 (7.4)
Other	95 (13.4)	69 (13.7)	26 (12.7)
Discharge diagnosis, No. (%)			
Bronchiolitis	436 (61.5)	278 (55.0)	158 (77.5)
Pneumonia	164 (23.1)	137 (27.1)	27 (13.2)
Bacterial pneumonia	86 (12.1)	76 (15.0)	10 (4.9)
Viral pneumonia	77 (10.9)	60 (11.9)	17 (8.3)
Asthma exacerbation	66 (9.3)	56 (11.1)	10 (4.9)
Viral upper respiratory tract infection	27 (3.8)	24 (4.8)	3 (1.5)
Croup	4 (0.6)	3 (0.6)	1 (0.5)
Coinfection, No. (%)			
Viral coinfection^b^	184 (26.0)	140 (27.7)	44 (21.6)
Enterovirus or rhinovirus	72 (10.2)	54 (10.7)	18 (8.8)
Seasonal coronaviruses	32 (4.5)	20 (4.0)	12 (5.9)
Adenovirus	25 (3.5)	23 (4.6)	2 (1.0)
SARS-CoV-2	23 (3.2)	19 (3.8)	4 (2.0)
Influenza (A or B)	18 (2.5)	14 (2.8)	4 (2.0)
Parainfluenza (1, 2, 3, or 4)	18 (2.5)	13 (2.6)	5 (2.5)
Human metapneumovirus	12 (1.7)	11 (2.2)	1 (0.5)
Bocavirus	12 (1.7)	9 (1.8)	3 (1.5)
Bacterial coinfection	20 (2.8)	8 (1.6)	12 (5.9)

^a^ Patients may have had multiple symptoms at presentation, resulting in a sum greater than 100%.

^b^ Patients may have had multiple coinfections, resulting in a sum greater than the number of patients with coinfections.

In addition to RSV, 184 cases (26.0%) had a concurrent other respiratory virus identified, which included enterovirus or rhinovirus (72 [10.2%]), adenovirus (25 [3.5%]), SARS-CoV-2 (23 [3.2%]), and influenza A or B (18 [2.5%]) (Table 2). Bacterial coinfections were more common among patients with severe disease than among those with nonsevere disease (12 of 204 [5.9%] vs 8 of 505 [1.6%]).

### Outcomes

Outcomes of RSV-associated ARI hospitalizations, including those used to define the composite disease severity outcome, are described by age group in Table 3. Approximately one-fourth of cases (190 [26.8%]) required noninvasive ventilation, and 66 (9.3%) required invasive mechanical ventilation, of whom over half were younger than 3 months (noninvasive: 105 of 190 [55.3%] and invasive: 41 of 66 [62.1%]). One patient in the study cohort died. The median length of hospital stay was 3 days (IQR, 2-6 days). Approximately one-third of cases were admitted to the ICU (241 [34.0%]), over half of whom were younger than 3 months of age (123 of 241 [51.0%]). The median length of ICU stay was 3 days (IQR, 1-5 days). Complications such as need for chest tube insertion (9 [1.3%]), acute respiratory distress syndrome (6 [0.8%]), and septic shock (5 [0.7%]) were infrequent. There were 78 patients (11.0%) who had an unscheduled return visit to the emergency department within 30 days of discharge and 26 cases (3.7%) that required readmission to the hospital.

**Table 3.** Complications and Outcomes Among Children With Respiratory Syncytial Virus–Confirmed Acute Respiratory Tract Infections by Age

Characteristic	Total (N = 709)	Age group					
		<3 mo (n = 224)	3 to <6 mo (n = 53)	6 to <12 mo (n = 66)	12 to <24 mo (n = 109)	2 to <5 y (n = 183)	5 to <18 y (n = 74)
Primary outcome							
Disease severity, No. (%)							
Nonsevere disease	505 (71.2)	114 (50.9)	25 (47.2)	55 (83.3)	96 (88.1)	160 (87.4)	55 (74.3)
Severe disease	204 (28.8)	110 (49.1)	28 (52.8)	11 (16.7)	13 (11.9)	23 (12.6)	19 (25.7)
Secondary outcomes							
Highest level of care required, No. (%)							
General pediatric inpatient unit	468 (66.0)	101 (45.1)	26 (49.1)	50 (75.8)	91 (83.5)	149 (81.4)	51 (68.9)
PICU	241 (34.0)	123 (54.9)	27 (50.9)	16 (24.2)	18 (16.5)	34 (18.6)	23 (31.1)
Length of hospital stay, median (IQR), d	3.0 (2.0-6.0)	4.0 (3.0-7.0)	4.0 (3.0-6.0)	3.0 (1.0-4.0)	3.0 (2.0-4.0)	3.0 (2.0-5.0)	4.5 (2.0-7.0)
Length of PICU stay, median (IQR), d	3.0 (1.0-5.0)	4.0 (2.0-7.0)	2.0 (2.0-4.0)	1.0 (0.5-3.0)	2.0 (1.0-5.0)	2.0 (1.0-4.0)	3.0 (1.0-6.0)
Return visit to ED within 30 d, No. (%)	78 (11.0)	22 (9.8)	4 (7.5)	7 (10.6)	13 (11.9)	27 (14.8)	5 (6.8)
Readmission to hospital within 30 d, No. (%)	26 (3.7)	9 (4.0)	1 (1.9)	3 (4.5)	4 (3.7)	6 (3.3)	3 (4.1)
Respiratory or hemodynamic support required, No. (%)^a^							
Low-flow nasal cannula or mask	405 (57.1)	109 (48.7)	21 (39.6)	38 (57.6)	77 (70.6)	110 (60.1)	50 (67.6)
High-flow nasal cannula	371 (52.3)	154 (68.8)	39 (73.6)	33 (50.0)	46 (42.2)	73 (39.9)	26 (35.1)
Noninvasive ventilation (CPAP or BiPAP)	190 (26.8)	105 (46.9)	27 (50.9)	11 (16.7)	11 (10.1)	20 (10.9)	16 (21.6)
Invasive mechanical ventilation (intubation)	66 (9.3)	41 (18.3)	7 (13.2)	0	3 (2.8)	9 (4.9)	6 (8.1)
Vasoactive medication use	18 (2.5)	6 (2.7)	4 (7.5)	1 (1.5)	48 (44.0)	3 (1.6)	4 (5.4)
Severe complications, No. (%)							
Chest tube insertion	9 (1.3)	0	1 (1.9)	0	0	4 (2.2)	4 (5.4)
Acute respiratory distress syndrome	6 (0.8)	1 (0.4)	0	0	0	5 (2.7)	0
Septic shock	5 (0.7)	2 (0.9)	0	0	0	1 (0.5)	2 (2.7)
Extracorporeal membrane oxygenation	4 (0.6)	1 (0.4)	0	0	0	2 (1.1)	1 (1.4)
Pneumothorax	2 (0.3)	0	1 (1.9)	0	0	1 (0.5)	0
Kidney replacement therapy	2 (0.3)	0	0	0	0	1 (0.5)	1 (1.4)
Other^b^	5 (0.7)	2 (0.9)	0	0	0	2 (1.1)	1 (1.4)
Death during hospital admission, No. (%)	1 (0.1)	0	0	0	0	1 (0.5)	0

Abbreviations: BiPAP, bilevel positive airway pressure; CPAP, continuous positive airway pressure; ED, emergency department; PICU, pediatric intensive care unit.

^a^ Patients may have required multiple respiratory or hemodynamic supports, resulting in a sum greater than 100%.

^b^ Includes cardiac arrest (n = 2), empyema (n = 1), plasmapheresis (n = 1), and pneumomediastinum (n = 1).

### Risk Factors for Severe Outcomes

#### Severe Disease

**Figure 1.**
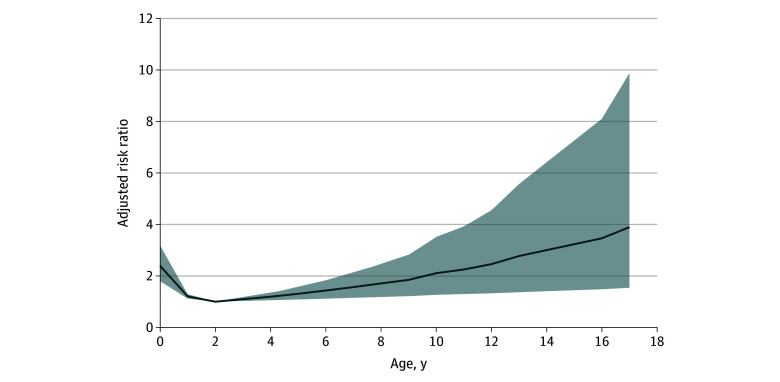
Association Between Age and Risk Ratio for Severe Disease Outcome Among Children Hospitalized With Respiratory Syncytial Virus (RSV)–Confirmed Acute Respiratory Tract Illness Graphical presentation of association between age and risk ratio for severe disease outcome among children hospitalized with RSV-confirmed acute respiratory tract illness when fit with restricted cubic spline and adjusted for sex, presence of any comorbidities, transfer from hospital, presence of any viral coinfection, symptom duration, and hospital site. Reference age is 2 years.

In the overall model, there was a nonlinear association between age and severe RSV infection (Figure 1), in which risk was highest among children who were very young, followed by a period of lower risk, then a subsequent increase in risk with age. Shorter duration of symptoms prior to presentation was associated with higher risk of severe disease (ARR, 0.95 [95% CI, 0.90-1.00] per 1-day increase) (eFigure 2 and eTable 1 in Supplement 1). We observed an effect modification whereby comorbidities were associated with increased risk among those aged 2 years or older but not those younger than 2 years.

**Figure 2.**
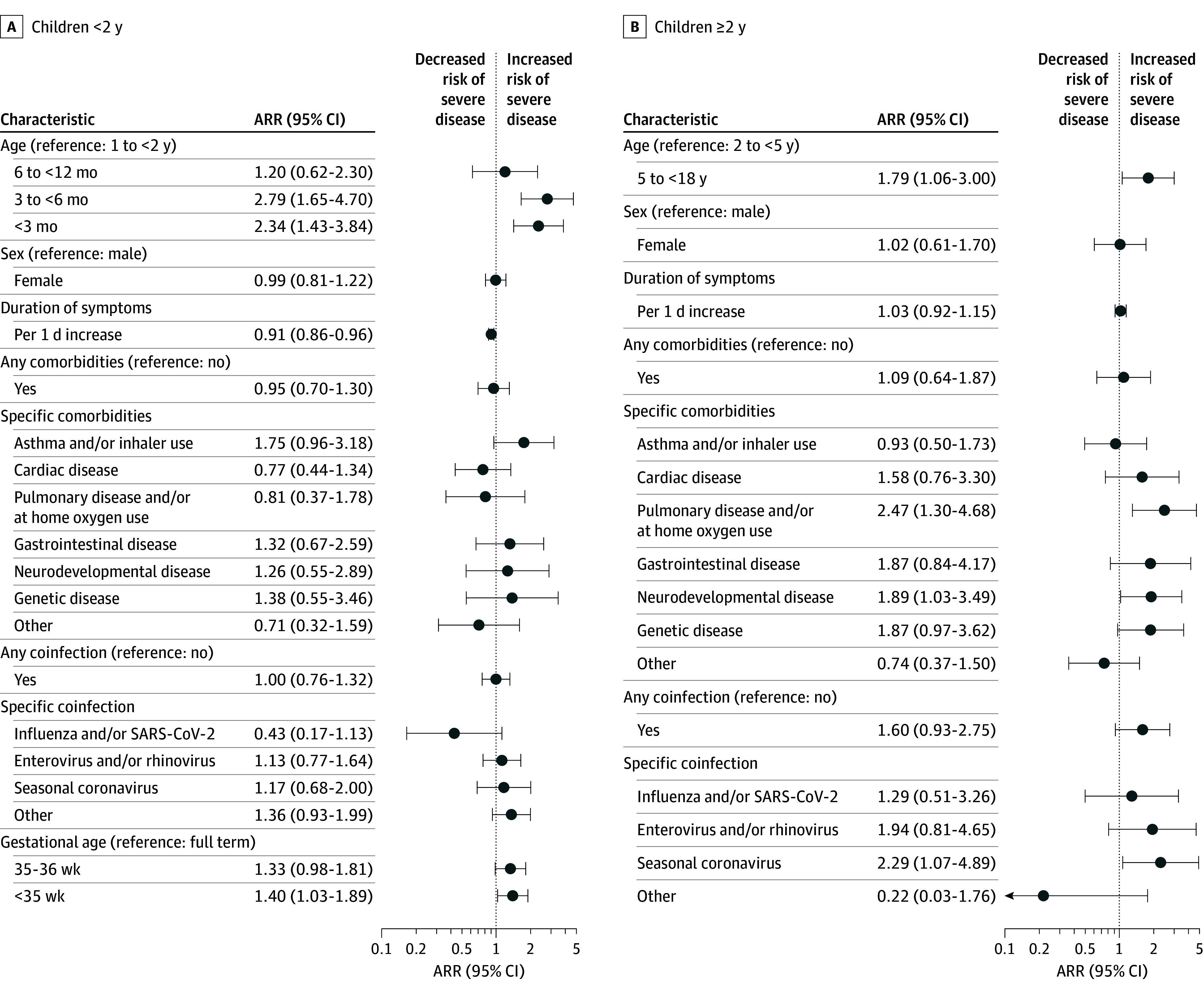
Forest Plot of Risk Factors for Severe Disease Among Children Hospitalized With Respiratory Syncytial Virus (RSV)–Confirmed Acute Respiratory Tract Illness Stratified by Age Groups ARR indicates adjusted risk ratio.

Figure 2A and B illustrate the findings from the age-stratified models. Among those younger than 2 years (eTable 2 in Supplement 1), those younger than 3 months (ARR, 2.34 [95% CI, 1.43-3.84]) and those aged 3 to less than 6 months (ARR, 2.79 [95% CI, 1.65-4.70]) both had increased risk of severe disease vs those aged 1 to less than 2 years. Premature infants younger than 35 weeks’ gestational age also had increased risk for severe disease (ARR, 1.40 [95% CI, 1.03-1.89]). For the comorbidities, the 95% CIs were too wide to draw clear conclusions.

Among those aged 2 years or older (eTable 3 in Supplement 1), the group aged 5 to less than 18 years had the highest risk of severe disease (ARR, 1.79 [95% CI, 1.06-3.00]) compared with the group aged 2 to 4 years. Pulmonary disease and use of home oxygen (ARR, 2.47 [95% CI, 1.30-4.68]) and neurologic, neuromuscular, and developmental conditions (ARR, 1.89 [95% CI, 1.03-3.49]) were associated with severe disease. Patients with seasonal coronavirus had a 2.29 times increased risk of severe disease (95% CI, 1.07-4.89).

#### ICU Admissions

Similar to findings with the severity outcome, shorter duration of symptoms (ARR, 0.95 [95% CI, 0.91-0.99]) was associated with higher risk of ICU admission (eFigure 3 and eTable 4 in Supplement 1). The age-stratified model for the outcome of ICU admission is displayed in eFigure 4 in Supplement 1. Among those younger than 2 years (eTable 5 in Supplement 1), both premature infants (<35 weeks’ gestational age) and late preterm infants (35-36 weeks’ gestational age) had an increased risk of ICU admission (<35 weeks: ARR, 1.29 [95% CI, 1.00-1.66]; 35-36 weeks: 1.48 [95% CI, 1.14-1.93]). Among those aged 2 years or older (eTable 6 in Supplement 1), comorbidities that incurred greater ICU admission risk included cardiac disease (ARR, 2.02 [95% CI, 1.13-3.60]) and pulmonary disease and/or home oxygen use (ARR, 3.01 [95% CI, 1.79-5.06]).

#### Length of Hospital Stay

Risk factors associated with a longer length of hospital stay included having a comorbidity (0.86 days [95% CI, 0.22-1.51 days]); neurologic, neuromuscular, or developmental diseases (2.18 days [95% CI, 1.16-3.20 days]); cardiac disease (1.19 days [95% CI, 0.25-2.13 days]); and gastrointestinal disease (2.72 days [95% CI, 1.66-3.79 days]) (eFigure 5 and eTable 7 in Supplement 1).

#### Severe Disease Among Nontransferred Patients

Compared with the main model for severe disease outcome (eFigure 2 in Supplement 1), the estimates were similar between the 2 models, with the 95% CIs largely overlapping (eFigure 6 and eTable 8 in Supplement 1). Duration of symptoms prior to hospital admission was no longer significantly associated with risk of severe disease among nontransfer patients (ARR, 0.87 [95% CI, 0.75-1.02]). In addition, cardiac disease and gastrointestinal disease were significantly associated with increased risk of severe disease (cardiac disease: ARR, 2.40 [95% CI, 1.02-5.63]; gastrointestinal disease: 2.61 [95% CI, 1.15-5.92]) among nontransfer patients.

## Discussion

The development of long-acting monoclonal antibodies for RSV infection, such as nirsevimab, warrants ensuring that risk criteria reflect current epidemiologic findings to maximize impact.^12^ Our study examined 709 children hospitalized with RSV-associated ARIs at 2 large, Canadian pediatric centers during the 2022-2023 respiratory viral season. Of these, 28.8% had severe illness, 34% required ICU admission, and 1 patient died. Severe disease occurred in all age groups, including previously healthy children and those with chronic conditions. Risk factors included younger age, prematurity, and, in older children, having pulmonary or neurologic, neuromuscular, or developmental conditions. Shorter symptom duration was associated with more severe disease. These findings shed light on the evolving clinical factors in pediatric RSV infections based on contemporary epidemiologic characteristics and will inform future efforts to reduce the burden of RSV infections.

In our study, the median age of patients was 13.1 months, with the highest proportion of RSV-associated ARIs among those younger than 3 months and those aged 2 to less than 5 years. Recent studies conducted after the COVID-19 pandemic have reported that RSV incidence has increased among older children, increasing the mean age of patients, which is a global phenomenon.^6,33,34,35,36,37,38,39^ This shift may stem from older children having prolonged susceptibility due to lack of prior exposure and waning maternal antibodies.^40,41,42^ In addition, potential SARS-CoV-2–induced immune dysregulation may have been associated with increased susceptibility to and severe outcomes from respiratory viruses in this population.^40,41,42^ Our findings highlight age as a key risk factor for severe disease, especially among children younger than 6 months and those older than 5 years. Although severe RSV disease has traditionally been associated with younger infants and our results suggest a need for continued focus on younger children, our findings highlight that prophylactic RSV treatments for older age groups, in which RSV-associated ARI prevalence and severity have also increased, may now be warranted.^7,8,25,26,28^

In our study, most RSV-positive children with ARIs lacked comorbidities, aligning with previous research in which only 16% to 22% of cases involved such conditions.^8,9,33,34^ Conditions such as cardiac, pulmonary, and genetic diseases as well as prematurity have been recognized as risk factors for severe RSV in previous studies, and similar patterns were seen in our cohort, particularly for children aged 2 years or older.^7,14,25,26,27,28,30,43^ For those younger than 2 years, age less than 6 months and prematurity were identified as risk factors for severe disease and ICU admission. Our findings support current recommendations across jurisdictions that preventive strategies be applied broadly to all infants, regardless of medical history, given that, among children younger than 2 years, comorbidities as a whole are not consistently associated with severe RSV disease. Patients with a history of prematurity may still warrant ongoing special attention, particularly with respect to their continuing risk beyond 2 years of life. In Spain, broad use of nirsevimab immunization for all infants younger than 6 months has already proven to be beneficial in reducing RSV-related hospitalizations, a positive change that, if similarly applied to a larger landscape, can result in significant changes across health care systems.^14,15,16,17,18^

In contrast, for children aged 2 years or older, comorbidities traditionally associated with severe RSV disease remained as risk factors for severe disease and ICU admission. Current international guidelines recommend RSV prophylaxis for children older than 1 year at high risk, but none extend beyond the second year of life.^14,16,17^ Our data suggest that an approach of targeted RSV prophylaxis with new agents (eg, nirsevimab) for high-risk groups, as previously done with palivizumab, could be beneficial if it is extended to older children who are not currently covered by existing recommendations.^44^

Coinfections have long been recognized to influence transmission dynamics and clinical outcomes, potentially altering the severity of infections. One study noted increased frequency of fever and leukocytosis in RSV infection plus coinfection with another respiratory virus, but this did not correlate with disease severity.^45^ In our study, only seasonal coronavirus was identified as an independent risk factor for severe RSV-associated ARI, specifically among children aged 2 years or older. Continued research into the role of coinfections is important to refine future risk assessments and treatment strategies.^46^ We also sought to explore coinfection with human metapneumovirus as a potential risk factor for severe disease^47,48^ given its similarities to RSV in causing bronchiolitis; however, our sample size did not allow for a conclusive analysis.

In our study, children with severe RSV disease more commonly presented with increased work of breathing, apnea, tachypnea, and cyanosis. Although several scoring systems have been developed to assess RSV infection severity based on clinical features, such as respiratory rate and wheeze,^49,50,51,52^ these systems are often underused in clinical practice due to their time-consuming and cumbersome nature. However, these scoring systems have primarily been developed and validated for younger children, with the oldest age cohort being 3 years of age. Given our findings that the proportion of RSV-associated ARI among older children remains elevated compared with pre–COVID-19 levels and that children aged 5 to less than 18 years are at increased risk of severe disease, there is a clear need for further research to develop and validate scoring tools tailored to this older age group.

### Limitations

This study has some limitations. First, it was conducted at 2 large tertiary care pediatric centers with a retrospective design, which may introduce bias from missing data. Second, the findings reflect primarily patients with more serious RSV presentations, as milder cases are often not admitted to the hospital, are managed at community hospitals, or may not undergo viral testing, potentially leading to an underestimation of RSV cases in the nonsevere group. Therefore, our results may not be generalizable to RSV cases with less severe infections. Furthermore, as the study was conducted at children’s hospitals, the cases classified in the nonsevere group and managed on the general pediatric wards may inherently be more severe but remain on the ward compared with community hospitals due to greater tolerance for significant illness and ease in accessing ICU support in the event of deterioration. Third, during the surge of ARIs, thresholds for admission to the ICU varied given bed shortages and resources. However, there are often resource pressures in hospitals during busy winter viral seasons. Future work evaluating hospitalized patients at a range of hospital types would improve generalizability. Fourth, medical records of included patients did not readily include information related to palivizumab prophylaxis, limiting our ability to assess whether prophylaxis mitigated severe disease in the traditionally identified high-risk comorbidity population of patients who were admitted to the hospital with RSV-associated ARIs. Fifth, as 41% of our cohort consisted of transfers from community hospitals, referral bias likely contributed to an underestimation of outcomes. It is possible that factors that did not reach significance in this study may still be relevant for estimating severe illness in the broader population of children with RSV.

## Conclusions

In this multicenter retrospective cohort study, severe RSV-associated ARI was observed across all age groups among children with or without comorbid conditions. For those aged 2 years or older, severe disease was more likely among children with pulmonary and neurologic, neuromuscular, or developmental conditions. For children younger than 2 years, severe disease risk was increased among infants younger than 6 months and among those with a history of prematurity in the absence of other comorbidities. As health systems move toward universal prevention in infancy, our study supports this approach and suggests the potential added benefits associated with monoclonal antibody therapies for children aged 2 years or older in high-risk groups. Continued surveillance of RSV-associated ARI in the postpandemic era will help determine whether these observed changes in patient epidemiologic characteristics are transient or indicative of a lasting change.

## Notes

Supplement 1. **eFigure 1.** Patient Flow Through the Study **eFigure 2.** Forest Plot of Risk Factors of Severe Disease Among Children Hospitalized With RSV-Confirmed Acute Respiratory Illness **eFigure 3.** Forest Plot of Risk Factors of PICU Admissions Among Children Hospitalized With RSV-Confirmed Acute Respiratory Illness **eFigure 4.** Forest Plot of Risk Factors of PICU Admissions Among Children Hospitalized With RSV-Confirmed Acute Respiratory Illness Stratified by Age Group **eFigure 5.** Forest Plot of Factors Associated With Length of Stay in Among Children Hospitalized With RSV-Confirmed Acute Respiratory Illness **eFigure 6.** Forest Plot of Factors Associated With Severe Disease Among Children Hospitalized With RSV-Confirmed Acute Respiratory Illness Among Nontransferred Patients **eTable 1.** Risk Ratios for Severe RSV-ARI by Sex, Age, Comorbidity, Hospital Transfer, Coinfection, Symptom Duration and Location (n=709) **eTable 2.** Risk Ratios for Severe RSV-ARI by Sex, Age, Comorbidity, Hospital Transfer, Coinfection, Symptom Duration, Gestational Age, Age, and Location (n=452) **eTable 3.** Risk Ratios for Severe RSV-ARI by Sex, Age, Comorbidity, Hospital Transfer, Coinfection, Symptom Duration and Location (n=257) **eTable 4.** Risk Ratios for ICU Admission by Sex, Age, Comorbidity, Hospital Transfer, Coinfection, Symptom Duration and Location (n= 709) **eTable 5.** Risk Ratios for ICU Admission by Sex, Age, Comorbidity, Hospital Transfer, Coinfection, Symptom Duration, Gestational Age, Age, and Location (n=452) **eTable 6.** Risk Ratios for ICU Admission by Sex, Age, Comorbidity, Hospital Transfer, Coinfection, Symptom Duration and Location (n=257) **eTable 7.** Risk Ratios for Increased Duration of Hospitalization for RSV-ARI by Sex, Age, Comorbidity, Hospital Transfer, Coinfection, Symptom Duration and Location (n= 709) **eTable 8.** Risk Ratios for Severe RSV-ARI by Sex, Age, Comorbidity, Coinfection, Symptom Duration and Location for Nontransferred Patients (n=418)

Supplement 2. **Nonauthor Collaborators.** READAPT-Kids Study Group Members

Supplement 3.
Data Sharing Statement
