# Supplementary material for: Risk Factors for Severe Disease Among Children Hospitalized With Respiratory Syncytial Virus
Source: JAMA Netw Open. 2025 Apr 11;8(4):e254666. doi: 10.1001/jamanetworkopen.2025.4666 (PMC11992603; doi:10.1001/jamanetworkopen.2025.4666)
Supplement: Supplement 1. — eFigure 1. Patient Flow Through the Study eFigure 2. Forest Plot of Risk Factors of Severe Disease Among Children Hospitalized With RSV-Confirmed Acute Respiratory Illness eFigure 3. Forest Plot of Risk Factors of PICU Admissions Among Children Hospitalized With RSV-Confirmed Acute Respiratory Illness eFigure 4. Forest Plot of Risk Factors of PICU Admissions Among Children Hospitalized With RSV-Confirmed Acute Respiratory Illness Stratified by Age Group eFigure 5. Forest Plot of Factors Associated With Length of Stay in Among Children Hospitalized With RSV-Confirmed Acute Respiratory Illness eFigure 6. Forest Plot of Factors Associated With Severe Disease Among Children Hospitalized With RSV-Confirmed Acute Respiratory Illness Among Nontransferred Patients eTable 1. Risk Ratios for Severe RSV-ARI by Sex, Age, Comorbidity, Hospital Transfer, Coinfection, Symptom Duration and Location (n=709) eTable 2. Risk Ratios for Severe RSV-ARI by Sex, Age, Comorbidity, Hospital Transfer, Coinfection, Symptom Duration, Gestational Age, Age, and Location (n=452) eTable 3. Risk Ratios for Severe RSV-ARI by Sex, Age, Comorbidity, Hospital Transfer, Coinfection, Symptom Duration and Location (n=257) eTable 4. Risk Ratios for ICU Admission by Sex, Age, Comorbidity, Hospital Transfer, Coinfection, Symptom Duration and Location (n= 709) eTable 5. Risk Ratios for ICU Admission by Sex, Age, Comorbidity, Hospital Transfer, Coinfection, Symptom Duration, Gestational Age, Age, and Location (n=452) eTable 6. Risk Ratios for ICU Admission by Sex, Age, Comorbidity, Hospital Transfer, Coinfection, Symptom Duration and Location (n=257) eTable 7. Risk Ratios for Increased Duration of Hospitalization for RSV-ARI by Sex, Age, Comorbidity, Hospital Transfer, Coinfection, Symptom Duration and Location (n= 709) eTable 8. Risk Ratios for Severe RSV-ARI by Sex, Age, Comorbidity, Coinfection, Symptom Duration and Location for Nontransferred Patients (n=418) [file jamanetwopen-e254666-s001.pdf]

Supplementary Online Content

Kirolos N, Mtaweh H, Datta RR, et al; READAPT-Kids study group members. Risk factors for severe disease among children hospitalized with respiratory syncytial virus. *JAMA Netw Open.* 2025;8(4):e254666.

doi:10.1001/jamanetworkopen.2025.4666

eFigure 1. Patient Flow Through the Study
eFigure 2. Forest Plot of Risk Factors of Severe Disease Among Children Hospitalized With RSV-Confirmed Acute Respiratory Illness
eFigure 3. Forest Plot of Risk Factors of PICU Admissions Among Children Hospitalized With RSV-Confirmed Acute Respiratory Illness
eFigure 4. Forest Plot of Risk Factors of PICU Admissions Among Children Hospitalized With RSV-Confirmed Acute Respiratory Illness Stratified by Age Group
eFigure 5. Forest Plot of Factors Associated With Length of Stay in Among Children Hospitalized With RSV-Confirmed Acute Respiratory Illness
eFigure 6. Forest Plot of Factors Associated With Severe Disease Among Children Hospitalized With RSV-Confirmed Acute Respiratory Illness Among Nontransferred Patients
eTable 1. Risk Ratios for Severe RSV-ARI by Sex, Age, Comorbidity, Hospital Transfer, Coinfection, Symptom Duration and Location (n=709)
eTable 2. Risk Ratios for Severe RSV-ARI by Sex, Age, Comorbidity, Hospital Transfer, Coinfection, Symptom Duration, Gestational Age, Age, and Location (n=452)
eTable 3. Risk Ratios for Severe RSV-ARI by Sex, Age, Comorbidity, Hospital Transfer, Coinfection, Symptom Duration and Location (n=257)
eTable 4. Risk Ratios for ICU Admission by Sex, Age, Comorbidity, Hospital Transfer, Coinfection, Symptom Duration and Location (n= 709)
eTable 5. Risk Ratios for ICU Admission by Sex, Age, Comorbidity, Hospital Transfer, Coinfection, Symptom Duration, Gestational Age, Age, and Location (n=452)
eTable 6. Risk Ratios for ICU Admission by Sex, Age, Comorbidity, Hospital Transfer, Coinfection, Symptom Duration and Location (n=257)
eTable 7. Risk Ratios for Increased Duration of Hospitalization for RSV-ARI by Sex, Age, Comorbidity, Hospital Transfer, Coinfection, Symptom Duration and Location (n= 709)
eTable 8. Risk Ratios for Severe RSV-ARI by Sex, Age, Comorbidity, Coinfection, Symptom Duration and Location for Nontransferred Patients (n=418)

This supplementary material has been provided by the authors to give readers additional information about their work.

© 2025 Kirolos N et al. *JAMA Network Open.*

eFigure 1. Patient Flow Through the Study

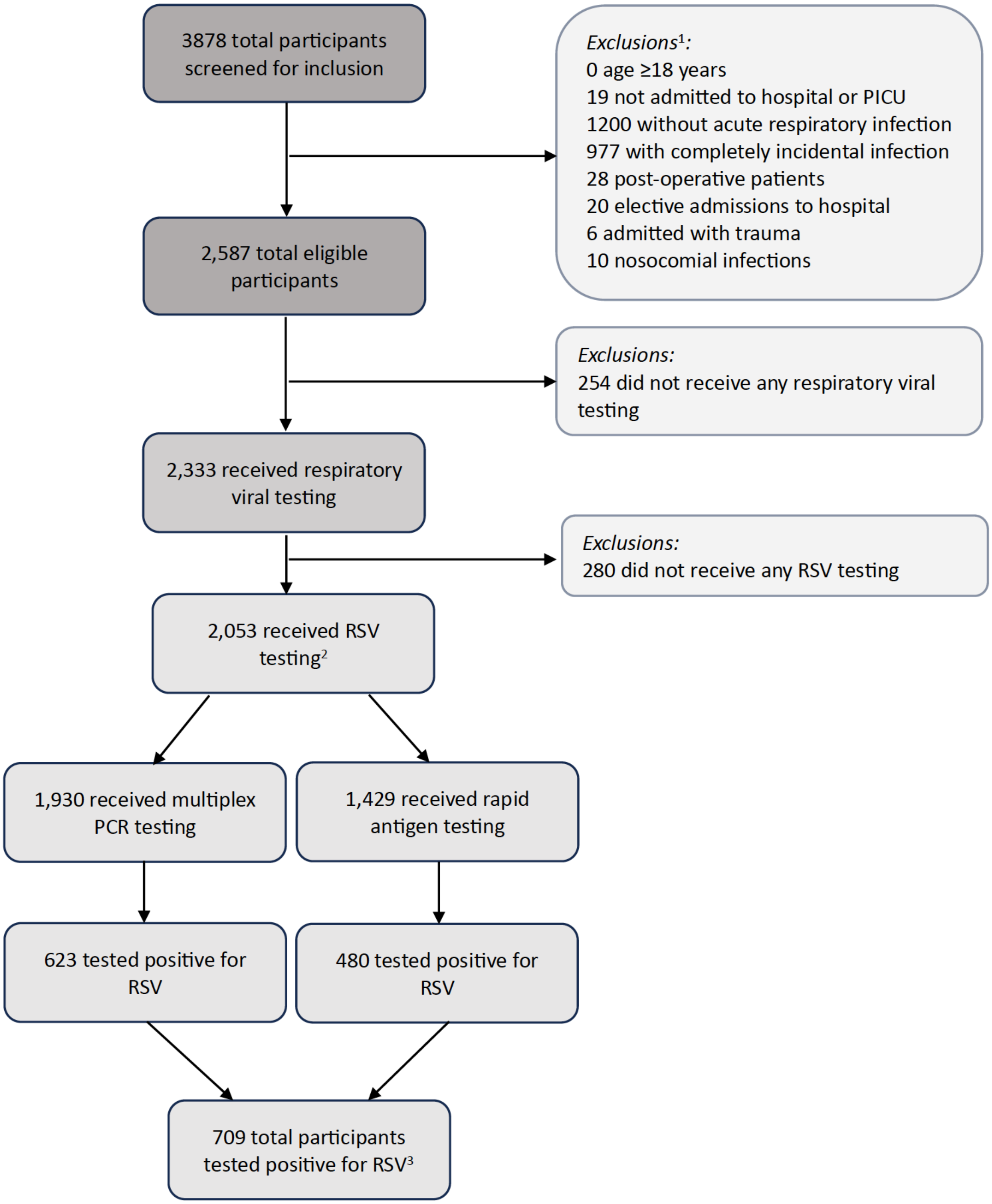

© 2025 Kirolos N et al. *JAMA Network Open.*

eFigure 2. Forest Plot of Risk Factors of Severe Disease Among Children Hospitalized With RSV-Confirmed Acute Respiratory Illness

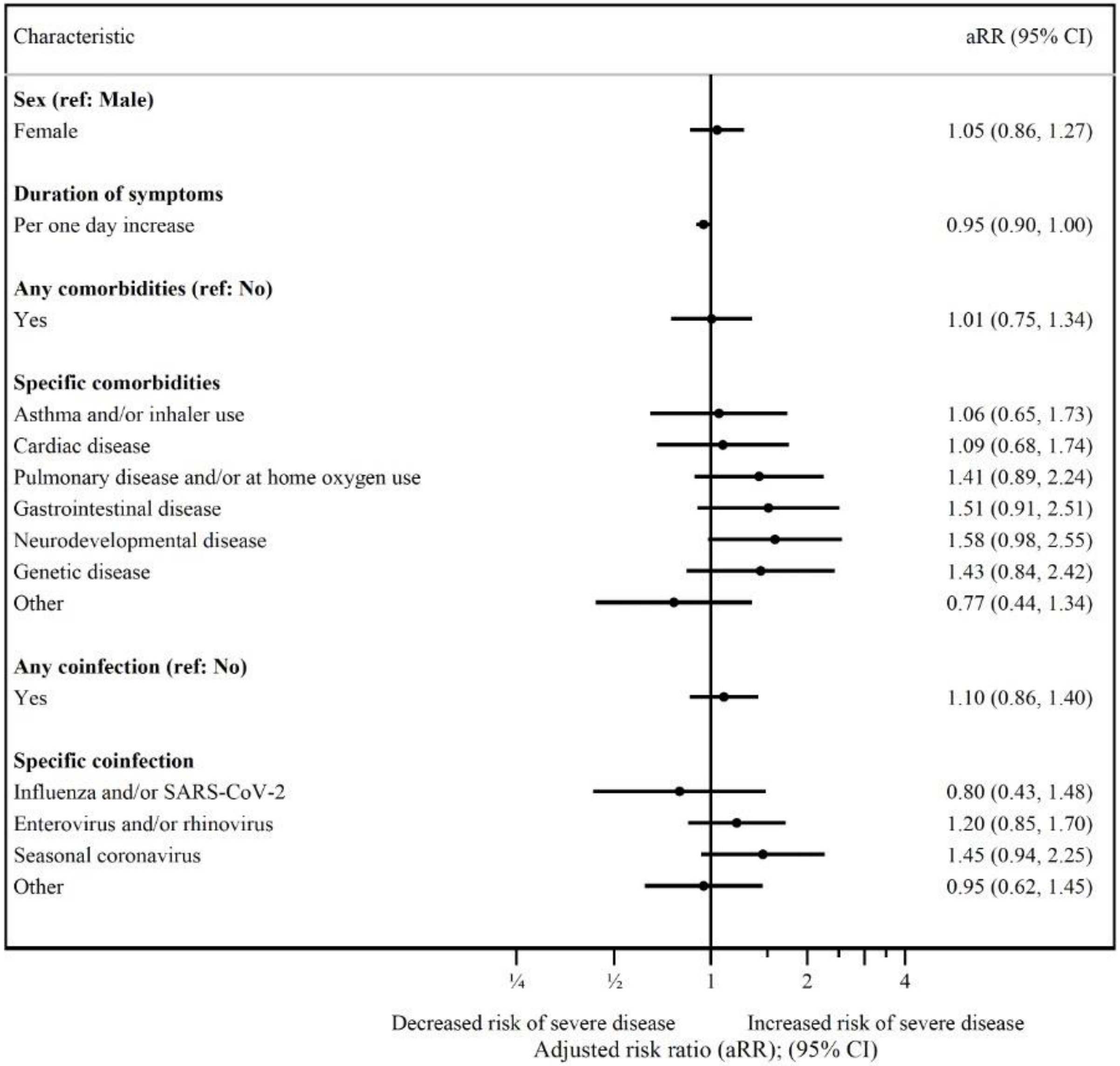

© 2025 Kirolos N et al. *JAMA Network Open.*

eFigure 3. Forest Plot of Risk Factors of PICU Admissions Among Children Hospitalized With RSV-Confirmed Acute Respiratory Illness

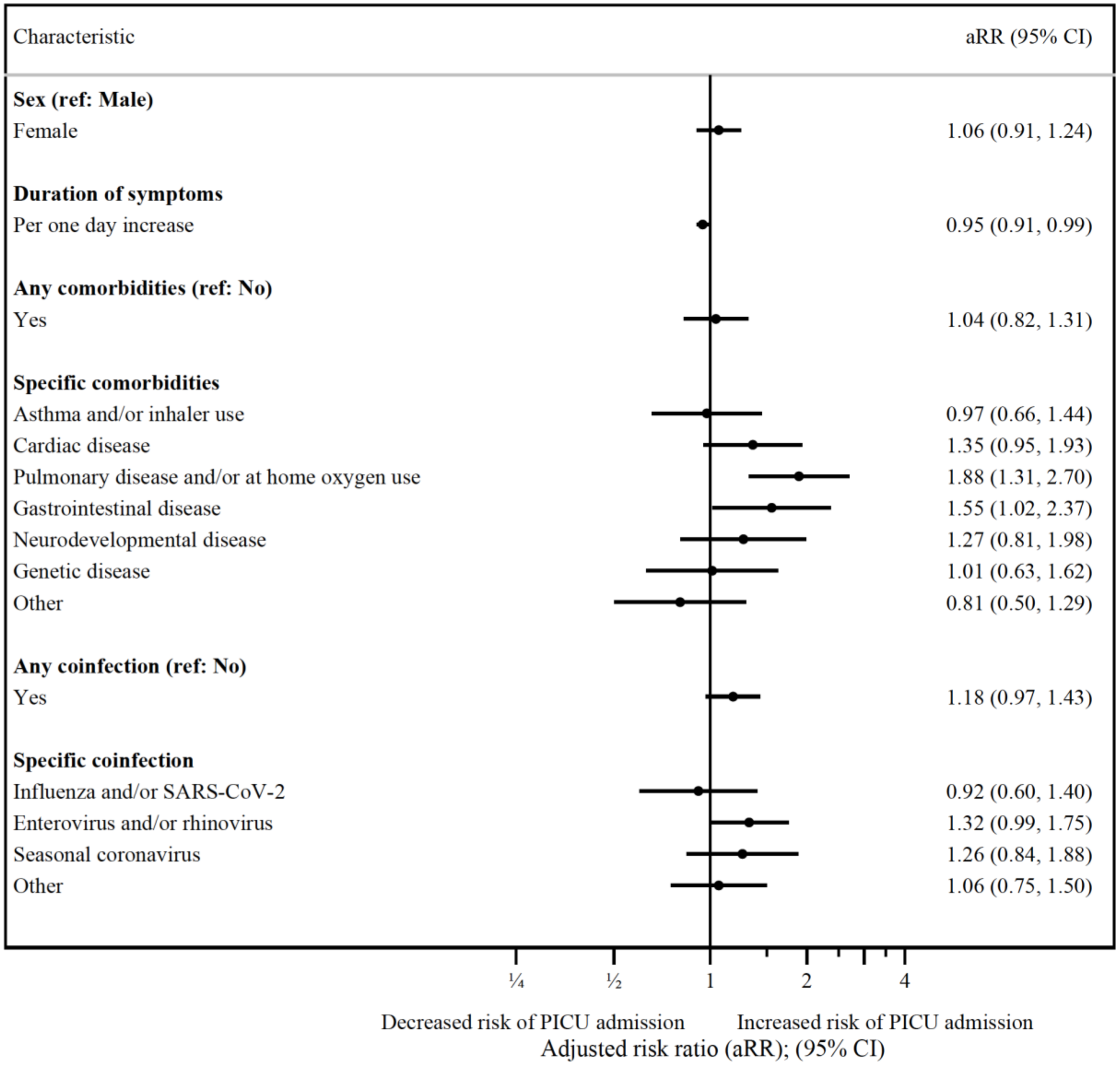

© 2025 Kirolos N et al. *JAMA Network Open.*

eFigure 4. Forest Plot of Risk Factors of PICU Admissions Among Children Hospitalized With RSV-Confirmed Acute Respiratory Illness Stratified by Age Group

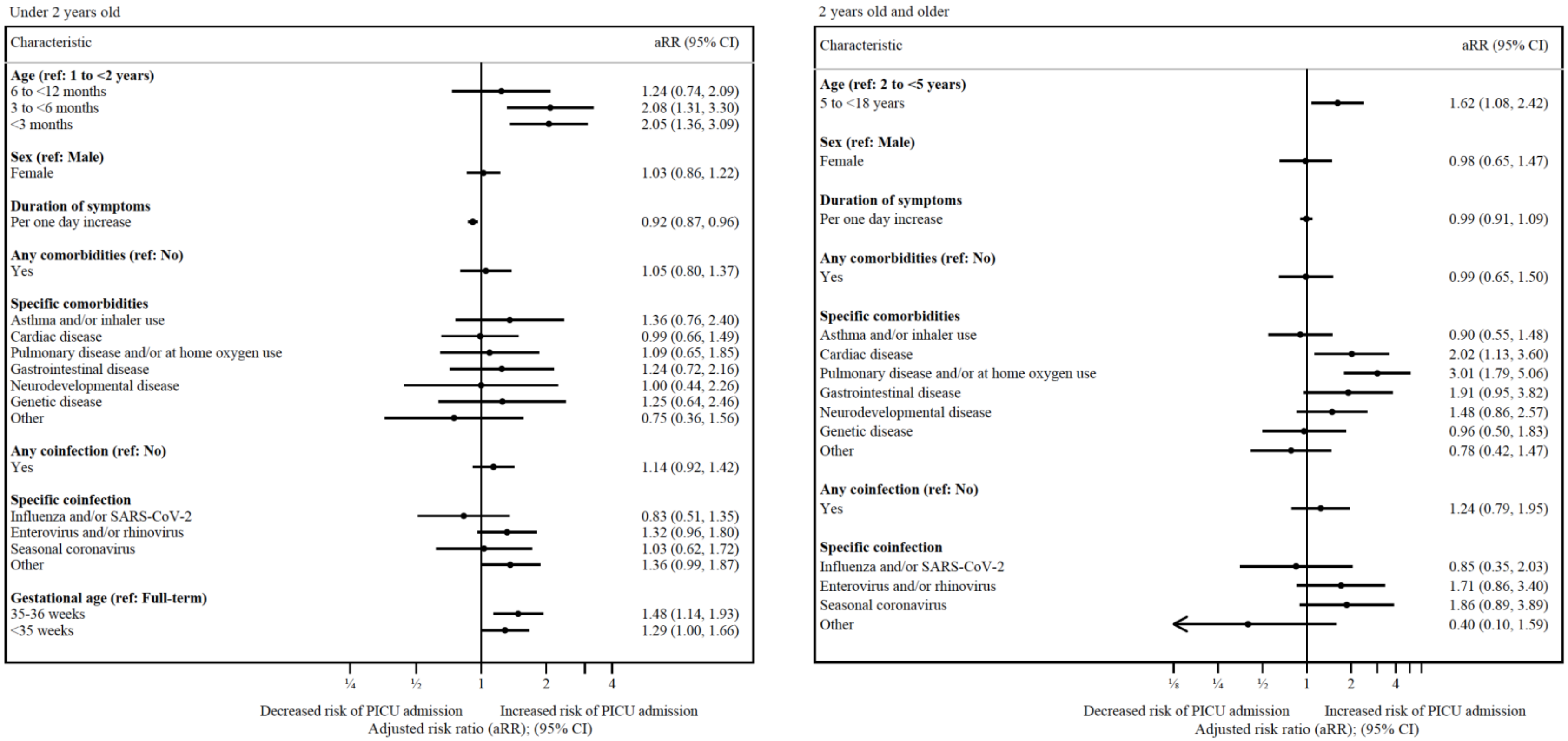

© 2025 Kirolos N et al. *JAMA Network Open.*

eFigure 5. Forest Plot of Factors Associated With Length of Stay in Among Children Hospitalized With RSV-Confirmed Acute Respiratory Illness

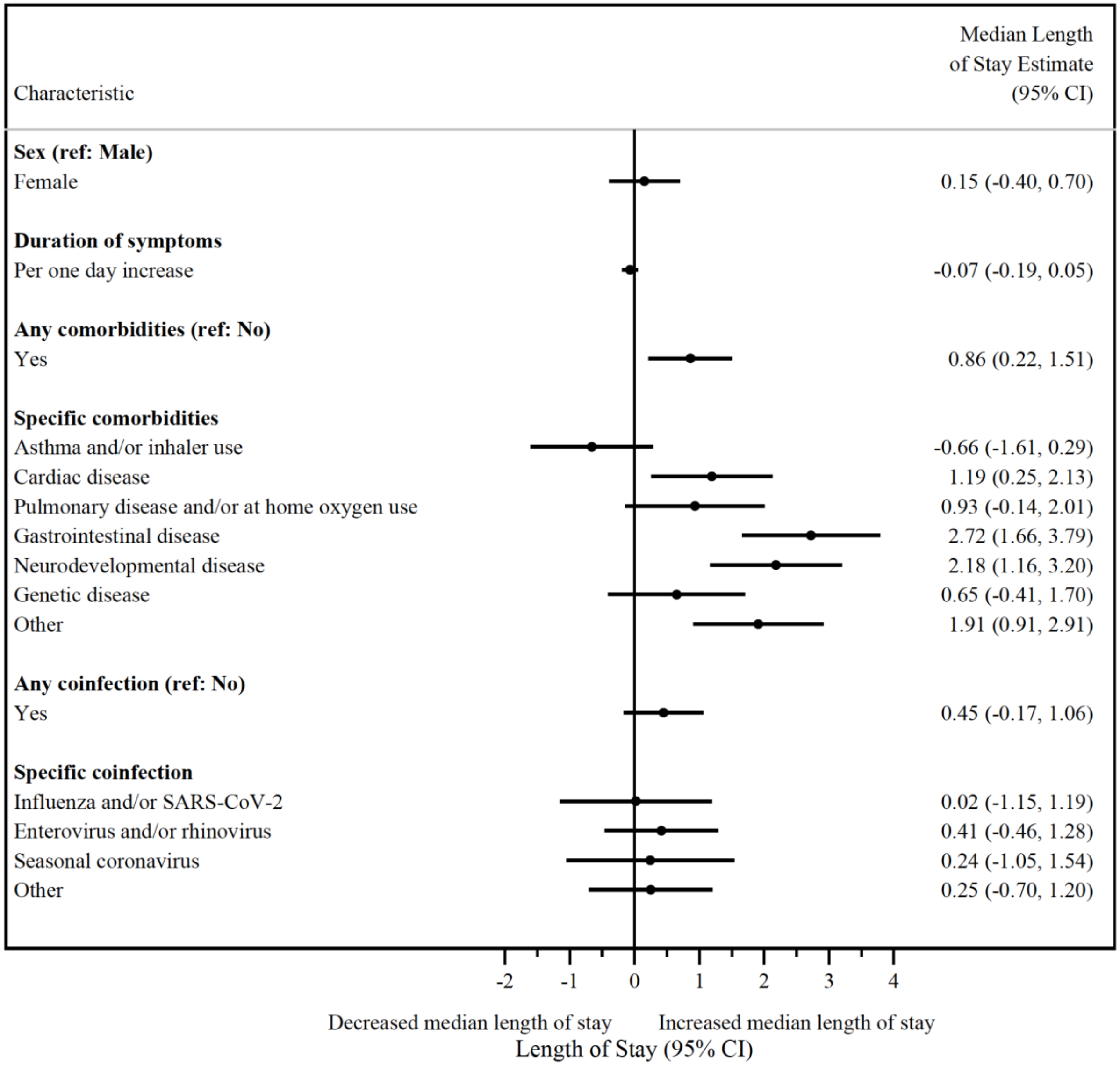

© 2025 Kirolos N et al. *JAMA Network Open.*

eFigure 6. Forest Plot of Factors Associated With Severe Disease Among Children Hospitalized With RSV-Confirmed Acute Respiratory Illness Among Nontransferred Patients

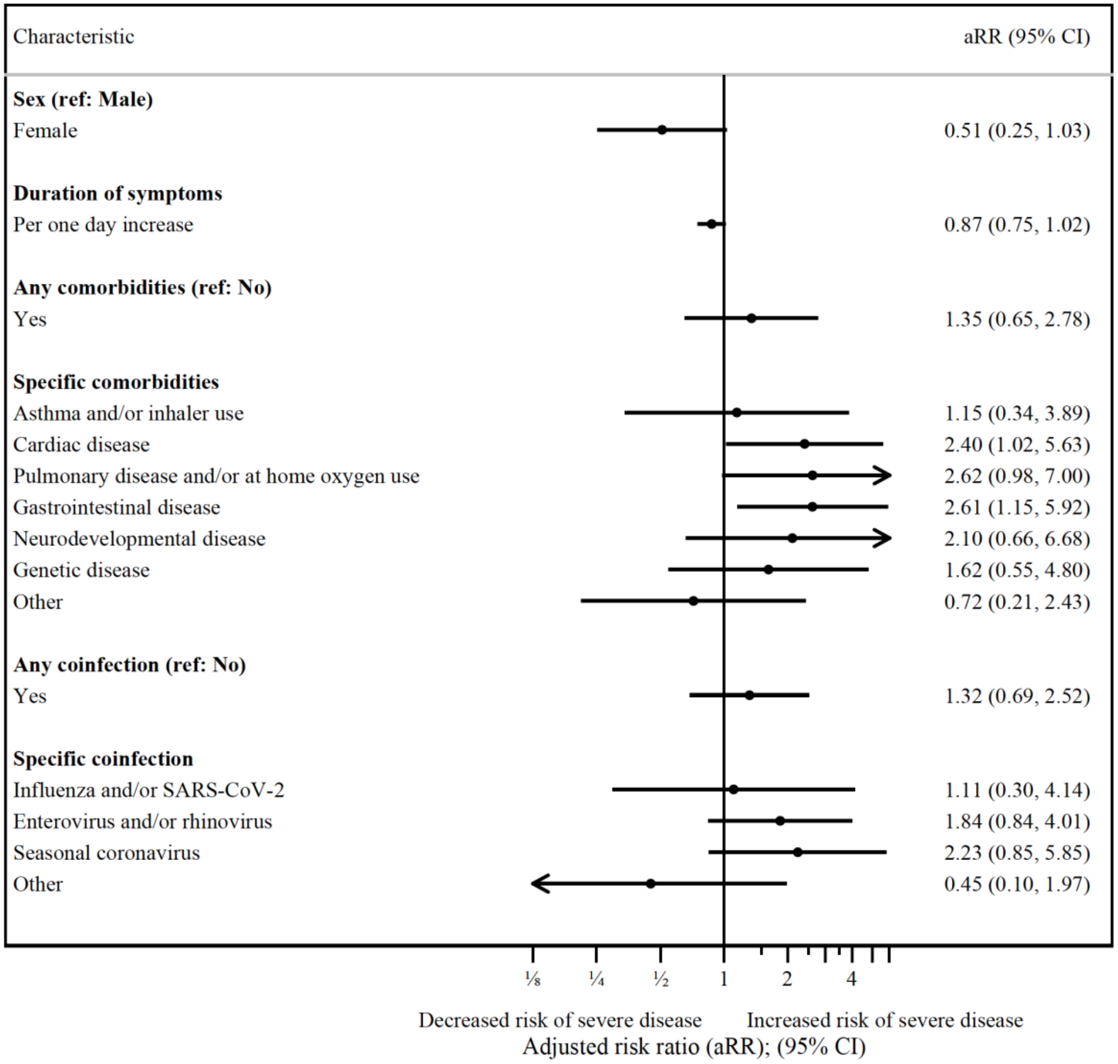

© 2025 Kirolos N et al. *JAMA Network Open.*

eTable 1. Risk Ratios for Severe RSV-ARI by Sex, Age, Comorbidity, Hospital Transfer, Coinfection, Symptom Duration and Location (n=709)

**Characteristics**	**Disease Severity, n (%)**		**Crude Model**		**Adjusted Model***	
	**Non-Severe (n=505)**	**Severe (n=204)**	**Risk Ratio (95% CI)**	**p**	**Risk Ratio (95% CI)**	**p**
**Sex**						
Male	313 (44.1)	129 (18.2)	Reference		Reference	
Female	192 (27.1)	75 (10.6)	0.96 (0.76, 1.22)	0.756	1.05 (0.86, 1.27)	0.643
**Hospital transfer [a]**						
No	381 (53.7)	37 (5.2)	Reference		Reference	
Yes	124 (17.5)	167 (23.6)	6.48 (4.69, 8.96)	<0.001	5.47 (3.92, 7.65)	<0.001
**Duration of symptoms [b] (days), median (IQR)**	4.0 (3.0, 5.0)	4.0 (2.0, 5.0)	0.92 (0.87, 0.98)	0.006	0.95 (0.90, 1.00)	0.043
**Any comorbidities**						
No	328 (46.3)	158 (22.3)	Reference		Reference	
Yes	177 (25.0)	46 (6.5)	0.63 (0.48, 0.85)	0.002	1.01 (0.75, 1.34)	0.971
**Asthma and/or inhaler use**						
No	457 (64.5)	189 (26.7)	Reference		Reference	
Yes	48 (6.8)	15 (2.1)	0.81 (0.51, 1.29)	0.378	1.06 (0.65, 1.73)	0.814
**Cardiac disease**						
No	456 (64.3)	190 (26.8)	Reference		Reference	
Yes	49 (6.9)	14 (2.0)	0.76 (0.47, 1.22)	0.250	1.09 (0.68, 1.74)	0.719
**Pulmonary disease and/or at home oxygen use**						
No	469 (66.1)	192 (27.1)	Reference		Reference	
Yes	36 (5.1)	12 (1.7)	0.86 (0.52, 1.43)	0.560	1.41 (0.89, 2.24)	0.141
**Gastrointestinal disease**						
No	468 (66.0)	193 (27.2)	Reference		Reference	
Yes	37 (5.2)	11 (1.6)	0.78 (0.46, 1.34)	0.373	1.51 (0.91, 2.51)	0.112

© 2025 Kirolos N et al. *JAMA Network Open.*

**Neurodevelopmental disease**						
No	464 (65.4)	189 (26.7)	Reference		Reference	
Yes	41 (5.8)	15 (2.1)	0.93 (0.59, 1.45)	0.736	1.58 (0.98, 2.55)	0.060
**Genetic disease**						
No	460 (64.9)	191 (26.9)	Reference		Reference	
Yes	45 (6.3)	13 (1.8)	0.76 (0.47, 1.25)	0.285	1.43 (0.84, 2.42)	0.187
**Other**						
No	452 (63.8)	194 (27.4)	Reference		Reference	
Yes	53 (7.5)	10 (1.4)	0.53 (0.30, 0.94)	0.031	0.77 (0.44, 1.34)	0.353
**Coinfection [c]**						
No	365 (51.5)	160 (22.6)	Reference		Reference	
Yes	140 (19.7)	44 (6.2)	0.78 (0.59, 1.05)	0.099	1.10 (0.86, 1.40)	0.448
**Influenza and/or SARS-CoV-2**						
No	474 (66.9)	196 (27.6)	Reference		Reference	
Yes	31 (4.4)	8 (1.1)	0.70 (0.37, 1.32)	0.269	0.80 (0.43, 1.48)	0.477
**Enterovirus and/or rhinovirus**						
No	451 (63.6)	186 (26.2)	Reference		Reference	
Yes	54 (7.6)	18 (2.5)	0.86 (0.56, 1.30)	0.467	1.20 (0.85, 1.70)	0.296
**Seasonal coronavirus**						
No	485 (68.4)	192 (27.1)	Reference		Reference	
Yes	20 (2.8)	12 (1.7)	1.32 (0.83, 2.10)	0.237	1.45 (0.94, 2.25)	0.096
**Other [d]**						
No	451 (63.6)	193 (27.2)	Reference		Reference	
Yes	54 (7.6)	11 (1.6)	0.56 (0.33, 0.98)	0.042	0.95 (0.62, 1.45)	0.810

*Adjusted risk ratios (aRR) were calculated using Poisson regression with robust standard errors. The overall model was adjusted for biological sex (binary), age (continuous, fit with 3 knots at 0.25, 1, & 2 years), comorbidity (binary), hospital transfer (binary), coinfection (binary), symptom duration (continuous), location (SickKids or BCCH). Sub-models for specific comorbidities/co-infections adjusted for the same, in addition to presence of other comorbidities/co-infections (binary).

[a] Patient transferred from another hospital, ICU, or ED

[b] Duration of symptoms attributable to respiratory infection prior to hospital admission

[c] Microbiologically-confirmed infection with one or more viral pathogens in addition to RSV

[d] Parainfluenza, adenovirus, bocavirus, metapneumovirus, and other

© 2025 Kirolos N et al. *JAMA Network Open.*

eTable 2. Risk Ratios for Severe RSV-ARI by Sex, Age, Comorbidity, Hospital Transfer, Coinfection, Symptom Duration, Gestational Age, Age, and Location (n=452)

Characteristics	Disease Severity, n (%)		Crude Model		Adjusted Model*	
	Non-Severe (n=290)	Severe (n=162)	Risk Ratio (95% CI)	p	Risk Ratio (95% CI)	p
**Sex**						
**Male**	185 (40.9)	104 (23.0)	Reference		Reference	
**Female**	105 (23.2)	58 (12.8)	0.99 (0.76, 1.28)	0.932	0.99 (0.81, 1.22)	0.941
**Hospital transfer [a]**						
No	212 (46.9)	24 (5.3)	Reference		Reference	
Yes	78 (17.3)	138 (30.5)	6.28 (4.24, 9.30)	<0.001	5.21 (3.45, 7.87)	<0.001
**Duration of symptoms [b] (days), median (IQR)**	4.0 (3.0, 6.0)	4.0 (2.0, 5.0)	0.89 (0.83, 0.95)	<0.001	0.91 (0.86, 0.96)	0.001
**Any comorbidities**						
No	226 (50.0)	140 (31.0)	Reference			
Yes	64 (14.2)	22 (4.9)	0.67 (0.46, 0.98)	0.040	0.95 (0.70, 1.30)	0.760
**Asthma and/or inhaler use**						
No	284 (62.8)	157 (34.7)	Reference		Reference	
Yes	6 (1.3)	5 (1.1)	1.28 (0.66, 2.47)	0.468	1.75 (0.96, 3.18)	0.068
**Cardiac disease**						
No	265 (58.6)	154 (34.1)	Reference		Reference	
Yes	25 (5.5)	8 (1.8)	0.66 (0.36, 1.22)	0.186	0.77 (0.44, 1.34)	0.353
**Pulmonary disease and/or at home oxygen use**						
No	275 (60.8)	159 (35.2)	Reference		Reference	
Yes	15 (3.3)	3 (0.7)	0.45 (0.16, 1.29)	0.138	0.81 (0.37, 1.78)	0.599
**Gastrointestinal disease**						
No	277 (61.3)	157 (34.7)	Reference		Reference	
Yes	13 (2.9)	5 (1.1)	0.77 (0.36, 1.64)	0.494	1.32 (0.67, 2.59)	0.420

© 2025 Kirolos N et al. *JAMA Network Open.*

**Neurodevelopmental disease**						
No	279 (61.7)	159 (35.2)	Reference		Reference	
Yes	11 (2.4)	3 (0.7)	0.59 (0.21, 1.62)	0.307	1.26 (0.55, 2.89)	0.584
**Genetic disease**						
No	276 (61.1)	158 (35.0)	Reference		Reference	
Yes	14 (3.1)	4 (0.9)	0.61 (0.25, 1.46)	0.268	1.38 (0.55, 3.46)	0.492
**Other**						
No	270 (59.7)	158 (35.0)	Reference		Reference	
Yes	20 (4.4)	4 (0.9)	0.45 (0.18, 1.12)	0.085	0.71 (0.32, 1.59)	0.406
**Coinfection [c]**						
No	211 (46.7)	132 (29.2)	Reference		Reference	
Yes	79 (17.5)	30 (6.6)	0.72 (0.51, 1.00)	0.049	1.00 (0.76, 1.32)	0.992
**Influenza and/or SARS-CoV-2**						
No	272 (60.2)	159 (35.2)	Reference		Reference	
Yes	18 (4.0)	3 (0.7)	0.39 (0.13, 1.11)	0.078	0.43 (0.17, 1.13)	0.086
**Enterovirus and/or rhinovirus**						
No	258 (57.1)	150 (33.2)	Reference		Reference	
Yes	32 (7.1)	12 (2.7)	0.74 (0.45, 1.22)	0.241	1.13 (0.77, 1.64)	0.541
**Seasonal coronavirus**						
No	277 (61.3)	154 (34.1)	Reference		Reference	
Yes	13 (2.9)	8 (1.8)	1.07 (0.61, 1.87)	0.823	1.17 (0.68, 2.00)	0.568
**Other [d]**						
No	266 (58.8)	152 (33.6)	Reference		Reference	
Yes	24 (5.3)	10 (2.2)	0.81 (0.47, 1.38)	0.438	1.36 (0.93, 1.99)	0.113
**Age**						
1 to <2 years	96 (21.2)	13 (2.9)	Reference		Reference	
6 to <12 months	55 (12.2)	11 (2.4)	1.40 (0.66, 2.94)	0.378	1.20 (0.62, 2.30)	0.589
3 to <6 months	25 (5.5)	28 (6.2)	4.43 (2.50, 7.84)	<0.001	2.79 (1.65, 4.70)	<0.001
<3 months	114 (25.2)	110 (24.3)	4.12 (2.43, 6.98)	<0.001	2.34 (1.43, 3.84)	0.001

© 2025 Kirolos N et al. *JAMA Network Open.*

**Gestational age**						
Full-term	235 (52.0)	125 (27.7)	Reference		Reference	
35-36 weeks	20 (4.4)	20 (4.4)	1.44 (1.02, 2.03)	0.036	1.33 (0.98, 1.81)	0.063
<35 weeks	20 (4.4)	16 (3.5)	1.28 (0.86, 1.89)	0.217	1.40 (1.03, 1.89)	0.030

*Adjusted risk ratios (aRR) were calculated using Poisson regression with robust standard errors. The overall model was adjusted for biological sex (binary), comorbidity (binary), hospital transfer (binary), coinfection (binary), symptom duration (continuous), gestational age (categorical), age (categorical), location (SickKids or BCCH). Sub-models for specific comorbidities/co-infections adjusted for same, in addition to presence of other comorbidities/co-infections (binary).
[a] Patient transferred from another hospital, ICU, or ED
[b] Duration of symptoms attributable to respiratory infection prior to hospital admission
[c] Microbiologically-confirmed infection with one or more viral pathogens in addition to RSV
[d] Parainfluenza, adenovirus, bocavirus, metapneumovirus, and other

© 2025 Kirolos N et al. *JAMA Network Open.*

eTable 3. Risk Ratios for Severe RSV-ARI by Sex, Age, Comorbidity, Hospital Transfer, Coinfection, Symptom Duration and Location (n=257)

**Characteristics**	**Disease Severity, n (%)**		**Crude Model**		**Adjusted Model***	
	**Non-Severe (n=215)**	**Severe (n=42)**	**Risk Ratio (95% CI)**	**p**	**Risk Ratio (95% CI)**	**p**
**Sex**						
**Male**	128 (49.8)	25 (9.7)	Reference		Reference	
**Female**	87 (33.9)	17 (6.6)	1.00 (0.57, 1.76)	0.999	1.02 (0.61, 1.70)	0.947
**Hospital transfer [a]**						
No	169 (65.8)	13 (5.1)	Reference		Reference	
Yes	46 (17.9)	29 (11.3)	5.41 (2.98, 9.84)	<0.001	5.94 (3.31, 10.66)	<0.001
**Duration of symptoms [b] (days), median (IQR)**	4.0 (3.0, 5.0)	4.0 (3.0, 7.0)	1.03 (0.91, 1.18)	0.627	1.03 (0.92, 1.15)	0.608
**Any comorbidities**						
No	102 (39.7)	18 (7.0)	Reference		Reference	
Yes	113 (44.0)	24 (9.3)	1.17 (0.67, 2.05)	0.588	1.09 (0.64, 1.87)	0.743
**Asthma and/or inhaler use**						
No	173 (67.3)	32 (12.5)	Reference		Reference	
Yes	42 (16.3)	10 (3.9)	1.23 (0.65, 2.34)	0.525	0.93 (0.50, 1.73)	0.821
**Cardiac disease**						
No	191 (74.3)	36 (14.0)	Reference		Reference	
Yes	24 (9.3)	6 (2.3)	1.26 (0.58, 2.74)	0.559	1.58 (0.76, 3.30)	0.222
**Pulmonary disease and/or at home oxygen use**						
No	194 (75.5)	33 (12.8)	Reference		Reference	
Yes	21 (8.2)	9 (3.5)	2.06 (1.10, 3.88)	0.025	2.47 (1.30, 4.68)	0.006
**Gastrointestinal disease**						
No	191 (74.3)	36 (14.0)	Reference		Reference	
Yes	24 (9.3)	6 (2.3)	1.26 (0.58, 2.74)	0.559	1.87 (0.84, 4.17)	0.128
**Neurodevelopmental disease**						
No	185 (72.0)	30 (11.7)	Reference		Reference	
Yes	30 (11.7)	12 (4.7)	2.05 (1.14, 3.67)	0.016	1.89 (1.03, 3.49)	0.040

© 2025 Kirolos N et al. *JAMA Network Open.*

**Genetic disease**						
No	184 (71.6)	33 (12.8)	Reference		Reference	
Yes	31 (12.1)	9 (3.5)	1.48 (0.77, 2.85)	0.242	1.87 (0.97, 3.62)	0.062
**Other**						
No	182 (70.8)	36 (14.0)	Reference		Reference	
Yes	33 (12.8)	6 (2.3)	0.93 (0.42, 2.06)	0.862	0.74 (0.37, 1.50)	0.405
**Coinfection** [c]						
No	154 (59.9)	28 (10.9)	Reference		Reference	
Yes	61 (23.7)	14 (5.4)	1.21 (0.68, 2.17)	0.516	1.60 (0.93, 2.75)	0.092
**Influenza and/or SARS-CoV-2**						
No	202 (78.6)	37 (14.4)	Reference		Reference	
Yes	13 (5.1)	5 (1.9)	1.79 (0.80, 4.01)	0.154	1.29 (0.51, 3.26)	0.596
**Enterovirus and/or rhinovirus**						
No	193 (75.1)	36 (14.0)	Reference		Reference	
Yes	22 (8.6)	6 (2.3)	1.36 (0.63, 2.95)	0.431	1.94 (0.81, 4.65)	0.137
**Seasonal coronavirus**						
No	208 (80.9)	38 (14.8)	Reference		Reference	
Yes	7 (2.7)	4 (1.6)	2.35 (1.02, 5.43)	0.045	2.29 (1.07, 4.89)	0.033
**Other** [d]						
No	185 (72.0)	41 (16.0)	Reference		Reference	
Yes	30 (11.7)	1 (0.4)	0.18 (0.03, 1.25)	0.083	0.22 (0.03, 1.76)	0.154
**Age (categorical)**						
2 to <5	160 (62.3)	23 (8.9)	Reference		Reference	
5 to <18	55 (21.4)	19 (7.4)	2.04 (1.18, 3.52)	0.010	1.79 (1.06, 3.00)	0.028

*Adjusted risk ratios (aRR) were calculated using Poisson regression with robust standard errors. The overall model was adjusted for biological sex (binary), age (categorical), comorbidity (binary), hospital transfer (binary), coinfection (binary), symptom duration (continuous), location (SickKids or BCCH). Sub-models for specific comorbidities/co-infections adjusted for same, in addition to presence of other comorbidities/co-infections (binary).

[a] Patient transferred from another hospital, ICU, or ED

[b] Duration of symptoms attributable to respiratory infection prior to hospital admission

[c] Microbiologically-confirmed infection with one or more viral pathogens in addition to RSV

[d] Parainfluenza, adenovirus, bocavirus, metapneumovirus, and other

© 2025 Kirolos N et al. *JAMA Network Open.*

eTable 4. Risk Ratios for ICU Admission by Sex, Age, Comorbidity, Hospital Transfer, Coinfection, Symptom Duration and Location (n= 709)

Characteristics	ICU Admission, n (%)		Crude Model		Adjusted Model*	
	No (n=468)	Yes (n=241)	Risk Ratio (95% CI)	p	Risk Ratio (95% CI)	p
**Sex**						
**Male**	291 (41.0)	151 (21.3)	Reference		Reference	
**Female**	177 (25.0)	90 (12.7)	0.99 (0.80, 1.22)	0.902	1.06 (0.91, 1.24)	0.462
**Hospital transfer** [a]						
No	374 (52.8)	44 (6.2)	Reference		Reference	
Yes	94 (13.3)	197 (27.8)	6.43 (4.81, 8.60)	<0.001	5.59 (4.15, 7.54)	<0.001
**Duration of symptoms** [b] **(days), median (IQR)**	4.0 (3.0, 5.5)	4.0 (2.0, 5.0)	0.92 (0.88, 0.97)	0.002	0.95 (0.91, 0.99)	0.011
**Any comorbidities**						
No	304 (42.9)	182 (25.7)	Reference		Reference	
Yes	164 (23.1)	59 (8.3)	0.71 (0.55, 0.90)	0.006	1.04 (0.82, 1.31)	0.740
**Asthma and/or inhaler use**						
No	423 (59.7)	223 (31.5)	Reference		Reference	
Yes	45 (6.3)	18 (2.5)	0.83 (0.55, 1.24)	0.360	0.97 (0.66, 1.44)	0.895
**Cardiac disease**						
No	425 (59.9)	221 (31.2)	Reference		Reference	
Yes	43 (6.1)	20 (2.8)	0.93 (0.64, 1.35)	0.698	1.35 (0.95, 1.93)	0.092
**Pulmonary disease and/or at home oxygen use**						
No	439 (61.9)	222 (31.3)	Reference		Reference	
Yes	29 (4.1)	19 (2.7)	1.18 (0.82, 1.70)	0.379	1.88 (1.31, 2.70)	0.001

© 2025 Kirolos N et al. *JAMA Network Open.*

**Gastrointestinal disease**						
No	434 (61.2)	227 (32.0)	Reference		Reference	
Yes	34 (4.8)	14 (2.0)	0.85 (0.54, 1.34)	0.480	1.55 (1.02, 2.37)	0.042
**Neurodevelopmental disease**						
No	428 (60.4)	225 (31.7)	Reference		Reference	
Yes	40 (5.6)	16 (2.3)	0.83 (0.54, 1.27)	0.391	1.27 (0.81, 1.98)	0.304
**Genetic disease**						
No	423 (59.7)	228 (32.2)	Reference		Reference	
Yes	45 (6.3)	13 (1.8)	0.64 (0.39, 1.05)	0.074	1.01 (0.63, 1.62)	0.960
**Other**						
No	418 (59.0)	228 (32.2)	Reference		Reference	
Yes	50 (7.1)	13 (1.8)	0.58 (0.36, 0.96)	0.034	0.81 (0.50, 1.29)	0.369
**Coinfection** [c]						
No	339 (47.8)	186 (26.2)	Reference		Reference	
Yes	129 (18.2)	55 (7.8)	0.84 (0.66, 1.08)	0.182	1.18 (0.97, 1.43)	0.102
**Influenza and/or SARS-CoV-2**						
No	440 (62.1)	230 (32.4)	Reference		Reference	
Yes	28 (3.9)	11 (1.6)	0.82 (0.49, 1.37)	0.452	0.92 (0.60, 1.40)	0.689
**Enterovirus and/or rhinovirus**						
No	419 (59.1)	218 (30.7)	Reference		Reference	
Yes	49 (6.9)	23 (3.2)	0.93 (0.66, 1.33)	0.703	1.32 (0.99, 1.75)	0.056
**Seasonal coronavirus**						
No	448 (63.2)	229 (32.3)	Reference		Reference	
Yes	20 (2.8)	12 (1.7)	1.11 (0.70, 1.76)	0.660	1.26 (0.84, 1.88)	0.263
**Other** [d]						

© 2025 Kirolos N et al. *JAMA Network Open.*

No	418 (59.0)	226 (31.9)	Reference		Reference	
Yes	50 (7.1)	15 (2.1)	0.66 (0.42, 1.04)	0.072	1.06 (0.75, 1.50)	0.734

*Adjusted risk ratios (aRR) were calculated using Poisson regression with robust standard errors. The overall model was adjusted for biological sex (binary), age (continuous, fit with 3 knots at 0.25, 1, & 2 years), comorbidity (binary), hospital transfer (binary), coinfection (binary), symptom duration (continuous), location (SickKids or BCCH). Sub-models for specific comorbidities/co-infections adjusted for same, in addition to presence of other comorbidities/co-infections (binary).

[a] Patient transferred from another hospital, ICU, or ED

[b] Duration of symptoms attributable to respiratory infection prior to hospital admission

[c] Microbiologically-confirmed infection with one or more viral pathogens in addition to RSV

[d] Parainfluenza, adenovirus, bocavirus, metapneumovirus, and other

© 2025 Kirolos N et al. *JAMA Network Open.*

eTable 5. Risk Ratios for ICU Admission by Sex, Age, Comorbidity, Hospital Transfer, Coinfection, Symptom Duration, Gestational Age, Age, and Location (n=452)

**Characteristics**	**ICU Admission, n (%)**		**Crude Model**		**Adjusted Model***	
	**No (n=268)**	**Yes (n=184)**	**Risk Ratio (95% CI)**	**p**	**Risk Ratio (95% CI)**	**p**
**Sex**						
**Male**	172 (38.1)	117 (25.9)	Reference		Reference	
**Female**	96 (21.2)	67 (14.8)	1.02 (0.81, 1.28)	0.897	1.03 (0.86, 1.22)	0.781
**Hospital transfer [a]**						
No	209 (46.2)	27 (6.0)	Reference		Reference	
Yes	59 (13.1)	157 (34.7)	6.35 (4.41, 9.15)	<0.001	5.48 (3.72, 8.07)	<0.001
**Duration of symptoms [b] (days), median (IQR)**	4.0 (3.0, 6.0)	4.0 (2.0, 5.0)	0.90 (0.85, 0.95)	<0.001	0.92 (0.87, 0.96)	<0.001
**Any comorbidities**						
No	210 (46.5)	156 (34.5)	Reference		Reference	
Yes	58 (12.8)	28 (6.2)	0.76 (0.55, 1.06)	0.106	1.05 (0.80, 1.37)	0.712
**Asthma and/or inhaler use**						
No	262 (58.0)	179 (39.6)	Reference		Reference	
Yes	6 (1.3)	5 (1.1)	1.12 (0.58, 2.16)	0.736	1.36 (0.76, 2.40)	0.298
**Cardiac disease**						
No	246 (54.4)	173 (38.3)	Reference		Reference	
Yes	22 (4.9)	11 (2.4)	0.81 (0.49, 1.33)	0.398	0.99 (0.66, 1.49)	0.960
**Pulmonary disease and/or at home oxygen use**						
No	255 (56.4)	179 (39.6)	Reference		Reference	
Yes	13 (2.9)	5 (1.1)	0.67 (0.32, 1.43)	0.304	1.09 (0.65, 1.85)	0.735
**Gastrointestinal disease**						
No	256 (56.6)	178 (39.4)	Reference		Reference	
Yes	12 (2.7)	6 (1.3)	0.81 (0.42, 1.58)	0.540	1.24 (0.72, 2.16)	0.436
**Neurodevelopmental disease**						

© 2025 Kirolos N et al. *JAMA Network Open.*

No	257 (56.9)	181 (40.0)	Reference		Reference	
Yes	11 (2.4)	3 (0.7)	0.52 (0.19, 1.42)	0.203	1.00 (0.44, 2.26)	1.000
**Genetic disease**						
No	255 (56.4)	179 (39.6)	Reference		Reference	
Yes	13 (2.9)	5 (1.1)	0.67 (0.32, 1.43)	0.304	1.25 (0.64, 2.46)	0.511
**Other**						
No	249 (55.1)	179 (39.6)	Reference		Reference	
Yes	19 (4.2)	5 (1.1)	0.50 (0.23, 1.10)	0.083	0.75 (0.36, 1.56)	0.443
**Coinfection** [c]						
No	198 (43.8)	145 (32.1)	Reference		Reference	
Yes	70 (15.5)	39 (8.6)	0.85 (0.64, 1.12)	0.244	1.14 (0.92, 1.42)	0.241
**Influenza and/or SARS-CoV-2**						
No	253 (56.0)	178 (39.4)	Reference		Reference	
Yes	15 (3.3)	6 (1.3)	0.69 (0.35, 1.37)	0.293	0.83 (0.51, 1.35)	0.455
**Enterovirus and/or rhinovirus**						
No	240 (53.1)	168 (37.2)	Reference		Reference	
Yes	28 (6.2)	16 (3.5)	0.88 (0.59, 1.33)	0.551	1.32 (0.96, 1.80)	0.084
**Seasonal coronavirus**						
No	255 (56.4)	176 (38.9)	Reference		Reference	
Yes	13 (2.9)	8 (1.8)	0.93 (0.53, 1.63)	0.807	1.03 (0.62, 1.72)	0.905
**Other** [d]						
No	247 (54.6)	171 (37.8)	Reference		Reference	
Yes	21 (4.6)	13 (2.9)	0.93 (0.60, 1.46)	0.765	1.36 (0.99, 1.87)	0.054
**Age**						
1 to <2 years	91 (20.1)	18 (4.0)	Reference		Reference	
6 to <12 months	50 (11.1)	16 (3.5)	1.47 (0.81, 2.68)	0.210	1.24 (0.74, 2.09)	0.418
3 to <6 months	26 (5.8)	27 (6.0)	3.08 (1.87, 5.08)	<0.001	2.08 (1.31, 3.30)	0.002
<3 months	101 (22.3)	123 (27.2)	3.33 (2.14, 5.16)	<0.001	2.05 (1.36, 3.09)	0.001
**Gestational age**						
Full-term	221 (48.9)	139 (30.8)	Reference		Reference	

© 2025 Kirolos N et al. *JAMA Network Open.*

35-36 weeks	15 (3.3)	25 (5.5)	1.62 (1.23, 2.13)	0.001	1.48 (1.14, 1.93)	0.004
<35 weeks	19 (4.2)	17 (3.8)	1.22 (0.85, 1.77)	0.286	1.29 (1.00, 1.66)	0.051

Adjusted risk ratios (aRR) were calculated using Poisson regression with robust standard errors. The overall model was adjusted for biological sex (binary), comorbidity (binary), hospital transfer (binary), coinfection (binary), symptom duration (continuous), gestational age (categorical), age (categorical), location (SickKids or BCCH). Sub-models for specific comorbidities/co-infections adjusted for same, in addition to presence of other comorbidities/co-infections (binary).

[a] Patient transferred from another hospital, ICU, or ED

[b] Duration of symptoms attributable to respiratory infection prior to hospital admission

[c] Microbiologically-confirmed infection with one or more viral pathogens in addition to RSV

[d] Parainfluenza, adenovirus, bocavirus, metapneumovirus, and other

© 2025 Kirolos N et al. *JAMA Network Open.*

eTable 6. Risk Ratios for ICU Admission by Sex, Age, Comorbidity, Hospital Transfer, Coinfection, Symptom Duration and Location (n=257)

**Characteristics**	**ICU Admission, n (%)**		**Crude Model**		**Adjusted Model***	
	**No (n=200)**	**Yes (n=57)**	**Risk Ratio (95% CI)**	**p**	**Risk Ratio (95% CI)**	**p**
**Sex**						
**Male**	119 (46.3)	34 (13.2)	Reference		Reference	
**Female**	81 (31.5)	23 (8.9)	1.00 (0.62, 1.59)	0.984	0.98 (0.65, 1.47)	0.922
**Hospital transfer [a]**						
No	165 (64.2)	17 (6.6)	Reference		Reference	
Yes	35 (13.6)	40 (15.6)	5.71 (3.46, 9.42)	<0.001	5.62 (3.46, 9.13)	<0.001
**Duration of symptoms [b] (days), median (IQR)**	4.0 (3.0, 5.0)	4.0 (3.0, 7.0)	1.00 (0.89, 1.11)	0.964	0.99 (0.91, 1.09)	0.883
**Any comorbidities**						
No	94 (36.6)	26 (10.1)	Reference		Reference	
Yes	106 (41.2)	31 (12.1)	1.04 (0.66, 1.66)	0.854	0.99 (0.65, 1.50)	0.947
**Asthma and/or inhaler use**						
No	161 (62.6)	44 (17.1)	Reference		Reference	
Yes	39 (15.2)	13 (5.1)	1.16 (0.68, 2.00)	0.580	0.90 (0.55, 1.48)	0.686
**Cardiac disease**						
No	179 (69.6)	48 (18.7)	Reference		Reference	
Yes	21 (8.2)	9 (3.5)	1.42 (0.78, 2.59)	0.255	2.02 (1.13, 3.60)	0.017
**Pulmonary disease and/or at home oxygen use**						
No	184 (71.6)	43 (16.7)	Reference		Reference	
Yes	16 (6.2)	14 (5.4)	2.46 (1.54, 3.94)	<0.001	3.01 (1.79, 5.06)	<0.001
**Gastrointestinal disease**						
No	178 (69.3)	49 (19.1)	Reference		Reference	
Yes	22 (8.6)	8 (3.1)	1.24 (0.65, 2.35)	0.520	1.91 (0.95, 3.82)	0.068
**Neurodevelopmental disease**						
No	171 (66.5)	44 (17.1)	Reference		Reference	
Yes	29 (11.3)	13 (5.1)	1.51 (0.90, 2.55)	0.122	1.48 (0.86, 2.57)	0.160

© 2025 Kirolos N et al. *JAMA Network Open.*

**Genetic disease**						
No	168 (65.4)	49 (19.1)	Reference		Reference	
Yes	32 (12.5)	8 (3.1)	0.89 (0.45, 1.73)	0.722	0.96 (0.50, 1.83)	0.893
**Other**						
No	169 (65.8)	49 (19.1)	Reference		Reference	
Yes	31 (12.1)	8 (3.1)	0.91 (0.47, 1.78)	0.788	0.78 (0.42, 1.47)	0.443
**Coinfection [c]**						
No	141 (54.9)	41 (16.0)	Reference		Reference	
Yes	59 (23.0)	16 (6.2)	0.95 (0.57, 1.58)	0.835	1.24 (0.79, 1.95)	0.347
**Influenza and/or SARS-CoV-2**						
No	187 (72.8)	52 (20.2)	Reference		Reference	
Yes	13 (5.1)	5 (1.9)	1.28 (0.58, 2.80)	0.542	0.85 (0.35, 2.03)	0.707
**Enterovirus and/or rhinovirus**						
No	179 (69.6)	50 (19.5)	Reference		Reference	
Yes	21 (8.2)	7 (2.7)	1.15 (0.58, 2.28)	0.700	1.71 (0.86, 3.40)	0.129
**Seasonal coronavirus**						
No	193 (75.1)	53 (20.6)	Reference		Reference	
Yes	7 (2.7)	4 (1.6)	1.69 (0.74, 3.83)	0.210	1.86 (0.89, 3.89)	0.097
**Other [d]**						
No	171 (66.5)	55 (21.4)	Reference		Reference	
Yes	29 (11.3)	2 (0.8)	0.27 (0.07, 1.04)	0.056	0.40 (0.10, 1.59)	0.194
**Age (categorical)**						
2 to <5	149 (58.0)	34 (13.2)	Reference		Reference	
5 to <18	51 (19.8)	23 (8.9)	1.67 (1.06, 2.64)	0.027	1.62 (1.08, 2.42)	0.020

*Adjusted risk ratios (aRR) were calculated using Poisson regression with robust standard errors.The overall model was adjusted for biological sex (binary), age (categorical), comorbidity (binary), hospital transfer (binary), coinfection (binary), symptom duration (continuous), location (SickKids or BCCH). Sub-models for specific comorbidities/co-infections adjusted for same, in addition to presence of other comorbidities/co-infections (binary).

[a] Patient transferred from another hospital, ICU, or ED

[b] Duration of symptoms attributable to respiratory infection prior to hospital admission

[c] Microbiologically-confirmed infection with one or more viral pathogens in addition to RSV

[d] Parainfluenza, adenovirus, bocavirus, metapneumovirus, and other

© 2025 Kirolos N et al. *JAMA Network Open.*

eTable 7. Risk Ratios for Increased Duration of Hospitalization for RSV-ARI by Sex, Age, Comorbidity, Hospital Transfer, Coinfection, Symptom Duration and Location (n= 709)

Characteristics	Length of Stay, median (IQR)	Crude Model		Adjusted Model*	
	n=709	Median Length of Stay (95% CI)	p	Median Length of Stay (95% CI)	p
**Sex**					
**Male**	4.0 (2.0, 6.0)	Reference		Reference	
**Female**	4.0 (2.0, 6.0)	0.00 (-0.70, 0.70)	>0.999	0.15 (-0.40, 0.70)	0.586
**Hospital transfer [a]**					
No	3.0 (2.0, 5.0)	Reference		Reference	
Yes	5.0 (3.0, 8.0)	2.00 (1.37, 2.63)	<0.001	2.49 (1.92, 3.07)	<0.001
**Duration of symptoms [b] (days), median (IQR)**	4.0 (3.0, 5.0)	-0.14 (-0.27, -0.01)	0.031	-0.07 (-0.19, 0.05)	0.257
**Any comorbidities**					
No	4.0 (2.0, 6.0)	Reference		Reference	
Yes	4.0 (2.0, 7.0)	0.00 (-0.59, 0.59)	>0.999	0.86 (0.22, 1.51)	0.009
**Asthma and/or inhaler use**					
No	4.0 (2.0, 6.0)	Reference		Reference	
Yes	3.0 (1.0, 7.0)	-1.00 (-2.19, 0.19)	0.099	-0.66 (-1.61, 0.29)	0.171
**Cardiac disease**					
No	4.0 (2.0, 6.0)	Reference		Reference	
Yes	5.0 (3.0, 8.0)	1.00 (-0.19, 2.19)	0.099	1.19 (0.25, 2.13)	0.013
**Pulmonary disease and/or at home oxygen use**					
No	4.0 (2.0, 6.0)	Reference		Reference	
Yes	5.0 (3.0, 8.5)	1.00 (-0.35, 2.35)	0.145	0.93 (-0.14, 2.01)	0.090
**Gastrointestinal disease**					
No	4.0 (2.0, 6.0)	Reference		Reference	

© 2025 Kirolos N et al. *JAMA Network Open.*

Yes	6.0 (3.0, 9.5)	2.00 (1.24, 2.76)	<0.001	2.72 (1.66, 3.79)	<0.001
**Neurodevelopmental disease**					
No	4.0 (2.0, 6.0)	Reference		Reference	
Yes	6.0 (3.0, 10.5)	2.00 (0.80, 3.20)	0.001	2.18 (1.16, 3.20)	<0.001
**Genetic disease**					
No	4.0 (2.0, 6.0)	Reference		Reference	
Yes	5.0 (2.0, 8.0)	1.00 (-0.23, 2.23)	0.112	0.65 (-0.41, 1.70)	0.228
**Other**					
No	4.0 (2.0, 6.0)	Reference		Reference	
Yes	5.0 (2.0, 8.0)	1.00 (-0.19, 2.19)	0.099	1.91 (0.91, 2.91)	<0.001
**Coinfection** [c]					
No	4.0 (2.0, 6.0)	Reference		Reference	
Yes	4.0 (2.0, 6.0)	0.00 (-0.77, 0.77)	>0.999	0.45 (-0.17, 1.06)	0.155
**Influenza and/or SARS-CoV-2**					
No	4.0 (2.0, 6.0)	Reference		Reference	
Yes	5.0 (2.0, 7.0)	1.00 (-0.48, 2.48)	0.186	0.02 (-1.15, 1.19)	0.974
**Enterovirus and/or rhinovirus**					
No	4.0 (2.0, 6.0)	Reference		Reference	
Yes	3.0 (2.0, 6.0)	-1.00 (-2.12, 0.12)	0.080	0.41 (-0.46, 1.28)	0.353
**Seasonal coronavirus**					
No	4.0 (2.0, 6.0)	Reference		Reference	
Yes	4.0 (2.0, 6.5)	0.00 (-1.59, 1.59)	>0.999	0.24 (-1.05, 1.54)	0.715
**Other** [d]					
No	4.0 (2.0, 6.0)	Reference		Reference	
Yes	3.0 (2.0, 6.0)	-1.00 (-2.17, 0.17)	0.094	0.25 (-0.70, 1.20)	0.606

© 2025 Kirolos N et al. *JAMA Network Open.*

*Median regression in days were calculated using Poisson regression with robust standard errors. The overall model was adjusted for biological sex (binary), age (continuous, fit with 3 knots at 0.25, 1, & 2 years), comorbidity (binary), hospital transfer (binary), coinfection (binary), symptom duration (continuous), location (SickKids or BCCH). Sub-models for specific comorbidities/co-infections adjusted for same, in addition to presence of other comorbidities/co-infections (binary).
[a] Patient transferred from another hospital, ICU, or ED
[b] Duration of symptoms attributable to respiratory infection prior to hospital admission
[c] Microbiologically-confirmed infection with one or more viral pathogens in addition to RSV
[d] Parainfluenza, adenovirus, bocavirus, metapneumovirus, and other

© 2025 Kirolos N et al. *JAMA Network Open.*

eTable 8. Risk Ratios for Severe RSV-ARI by Sex, Age, Comorbidity, Coinfection, Symptom Duration and Location for Nontransferred Patients (n=418)

**Characteristics**	**Disease Severity, n (%)**		**Crude Model**		**Adjusted Model***	
	Non-Severe (n=381)	Severe (n=37)	Risk Ratio (95% CI)	p	Risk Ratio (95% CI)	p
**Sex**						
**Male**	228 (54.5)	28 (6.7)	Reference		Reference	
**Female**	153 (36.6)	9 (2.2)	0.51 (0.25, 1.05)	0.067	0.51 (0.25, 1.03)	0.060
**Duration of symptoms [a] (days), median (IQR)**	4.0 (3.0, 6.0)	3.0 (2.0, 4.0)	0.85 (0.71, 1.02)	0.084	0.87 (0.75, 1.02)	0.083
**Any comorbidities**						
No	244 (58.4)	24 (5.7)	Reference		Reference	
Yes	137 (32.8)	13 (3.1)	0.97 (0.51, 1.85)	0.921	1.35 (0.65, 2.78)	0.421
**Asthma and/or inhaler use**						
No	350 (83.7)	34 (8.1)	Reference		Reference	
Yes	31 (7.4)	3 (0.7)	1.00 (0.32, 3.08)	0.995	1.15 (0.34, 3.89)	0.824
**Cardiac disease**						
No	341 (81.6)	31 (7.4)	Reference		Reference	
Yes	40 (9.6)	6 (1.4)	1.57 (0.69, 3.55)	0.284	2.40 (1.02, 5.63)	0.044
**Pulmonary disease and/or at home oxygen use**						
No	351 (84.0)	32 (7.7)	Reference		Reference	
Yes	30 (7.2)	5 (1.2)	1.71 (0.71, 4.11)	0.231	2.62 (0.98, 7.00)	0.056
**Gastrointestinal disease**						
No	347 (83.0)	32 (7.7)	Reference		Reference	
Yes	34 (8.1)	5 (1.2)	1.52 (0.63, 3.68)	0.354	2.61 (1.15, 5.92)	0.021
**Neurodevelopmental disease**						
No	346 (82.8)	32 (7.7)	Reference		Reference	
Yes	35 (8.4)	5 (1.2)	1.48 (0.61, 3.58)	0.388	2.10 (0.66, 6.68)	0.209
**Genetic disease**						

© 2025 Kirolos N et al. *JAMA Network Open.*

No	342 (81.8)	33 (7.9)	Reference		Reference	
Yes	39 (9.3)	4 (1.0)	1.06 (0.39, 2.84)	0.912	1.62 (0.55, 4.80)	0.383
**Other**						
No	337 (80.6)	34 (8.1)	Reference		Reference	
Yes	44 (10.5)	3 (0.7)	0.70 (0.22, 2.18)	0.535	0.72 (0.21, 2.43)	0.593
**Coinfection [b]**						
No	264 (63.2)	24 (5.7)	Reference		Reference	
Yes	117 (28.0)	13 (3.1)	1.20 (0.63, 2.28)	0.578	1.32 (0.69, 2.52)	0.403
**Influenza and/or SARS-CoV-2**						
No	361 (86.4)	35 (8.4)	Reference		Reference	
Yes	20 (4.8)	2 (0.5)	1.03 (0.26, 4.01)	0.968	1.11 (0.30, 4.14)	0.878
**Enterovirus and/or rhinovirus**						
No	334 (79.9)	30 (7.2)	Reference		Reference	
Yes	47 (11.2)	7 (1.7)	1.57 (0.73, 3.41)	0.250	1.84 (0.84, 4.01)	0.125
**Seasonal coronavirus**						
No	363 (86.8)	33 (7.9)	Reference		Reference	
Yes	18 (4.3)	4 (1.0)	2.18 (0.85, 5.62)	0.106	2.23 (0.85, 5.85)	0.104
**Other [c]**						
No	333 (79.7)	35 (8.4)	Reference		Reference	
Yes	48 (11.5)	2 (0.5)	0.42 (0.10, 1.70)	0.224	0.45 (0.10, 1.97)	0.289

*Adjusted risk ratios (aRR) were calculated using Poisson regression with robust standard errors. The overall model was adjusted for biological sex (binary), age (continuous, fit with 3 knots at 0.25, 1, & 2 years), comorbidity (binary), coinfection (binary), symptom duration (continuous), location (SickKids or BCCH). Sub-models for specific comorbidities/co-infections adjusted for same, in addition to presence of other comorbidities/co-infections (binary).

[a] Duration of symptoms attributable to respiratory infection prior to hospital admission

[b] Microbiologically-confirmed infection with one or more viral pathogens in addition to RSV

[c] Parainfluenza, adenovirus, bocavirus, metapneumovirus, and other

© 2025 Kirolos N et al. *JAMA Network Open.*
