# Supplementary material for: Risk Factors for Severe Disease Among Children Hospitalized With Respiratory Syncytial Virus
Source: JAMA Netw Open. 2025 Apr 11;8(4):e254666. doi: 10.1001/jamanetworkopen.2025.4666 (PMC11992603; doi:10.1001/jamanetworkopen.2025.4666)
Supplement: Supplement 2. — Nonauthor Collaborators. READAPT-Kids Study Group Members [file jamanetwopen-e254666-s002.pdf]

Supplemental Online Content: Nonauthor Collaborators
*First name, last name, and suffix (if applicable) are required and will appear in PubMed.

***Group Name(s): READAPT-Kids study group members**

*First Name and Middle Initial(s)	*Last Name	*Suffix (eg, Jr, III)	Academic Degrees	Institution	Location (city, state/province, country)	Role or Contribution, eg, chair, principal investigator	Group (if more than 1 Group listed in the byline) and/or Subgroup (eg, Steering Committee)
Mei	Han		MSc	Children's Hospital of Eastern Ontari	Ottawa, Ontario, Canada	Data Analyst	READAPT-Kids
Nicholas	Barrowman		PhD	Children's Hospital of Eastern Ontari	Ottawa, Ontario, Canada	Statistician	READAPT-Kids
Gabriele	Zitikyte		MSc	Children's Hospital of Eastern Ontari	Ottawa, Ontario, Canada	Data Analyst	READAPT-Kids
Anya	Nair		BSc	The Hospital for Sick Children	Toronto, Ontario, Canada	Research Coordinator	READAPT-Kids
Nafisa	Anwar		BSc	The Hospital for Sick Children	Toronto, Ontario, Canada	Research Assistant	READAPT-Kids
Rizk	ElMadbak		BSc	The Hospital for Sick Children	Toronto, Ontario, Canada	Research Assistant	READAPT-Kids
Haniyyah	Mahmood		BSc	The Hospital for Sick Children	Toronto, Ontario, Canada	Research Assistant	READAPT-Kids
Kody	Wolfstadt		BSc, MSc	The Hospital for Sick Children	Toronto, Ontario, Canada	Research Summer Student/Medical Student	READAPT-Kids
Nilushi	de Silva		BSc	The Hospital for Sick Children	Toronto, Ontario, Canada	Research Summer Student/Medical Student	READAPT-Kids
Polina	Kyrychenko		BSc	The Hospital for Sick Children	Toronto, Ontario, Canada	Research Summer Student/Medical Student	READAPT-Kids
Shamama	Raza		BSc	The Hospital for Sick Children	Toronto, Ontario, Canada	Research Assistant	READAPT-Kids
Vincent	Flores		MD	The Hospital for Sick Children	Toronto, Ontario, Canada	Research Assistant	READAPT-Kids
Keane	Fuerte		BSc	The Hospital for Sick Children	Toronto, Ontario, Canada	Research Assistant	READAPT-Kids
Pardis	Noormohammadpour		PhD	The Hospital for Sick Children	Toronto, Ontario, Canada	Research Assistant	READAPT-Kids
Bayley	Levy		BSc	The Hospital for Sick Children	Toronto, Ontario, Canada	Research Assistant	READAPT-Kids
Hafsa	Azher		BSc	The Hospital for Sick Children	Toronto, Ontario, Canada	Research Assistant	READAPT-Kids
Tom	McLaughlin		MPP, MD, FRCPC, FAAI	BC Children's Hospital	Vancouver, British Columbia, Canada	Co-Investigator	READAPT-Kids
Matt	Carwana		BSc, MD	BC Children's Hospital	Vancouver, British Columbia, Canada	Co-Investigator	READAPT-Kids
Srinivas	Murthy		MD	BC Children's Hospital	Vancouver, British Columbia, Canada	Co-Investigator	READAPT-Kids
Jenny	Retallack		BSc, MD, FRCPC	BC Children's Hospital	Vancouver, British Columbia, Canada	Co-Investigator	READAPT-Kids
Dave	Goldfarb		MD, FRCPC	BC Children's Hospital	Vancouver, British Columbia, Canada	Co-Investigator	READAPT-Kids
Candice	Wiedman		BSc, MSc	BC Children's Hospital	Vancouver, British Columbia, Canada	Project Manager	READAPT-Kids
Melissa	Braschel		MSc	BC Children's Hospital	Vancouver, British Columbia, Canada	Data Analyst	READAPT-Kids
Alam	Lakhani		MSc	BC Children's Hospital	Vancouver, British Columbia, Canada	Research Coordinator	READAPT-Kids
Opninder	Lindstrom		RRT	BC Children's Hospital	Vancouver, British Columbia, Canada	Research Coordinator, Registered Respiratory Therapist	READAPT-Kids
Sanja	Hadzi-Nikolova		BSc	BC Children's Hospital	Vancouver, British Columbia, Canada	Research Assistant/Nursing Student	READAPT-Kids
Min-Jung	Kim		BHSc	BC Children's Hospital	Vancouver, British Columbia, Canada	Research Student/Medical Student	READAPT-Kids
Victoria	Tapics		RRT	BC Children's Hospital	Vancouver, British Columbia, Canada	Research Assistant, Registered Respiratory Therapist	READAPT-Kids
Henry	Okpaladigbo		BA	BC Children's Hospital	Vancouver, British Columbia, Canada	Research Assistant	READAPT-Kids

Supplemental Online Content: Nonauthor Collaborators
*First name, last name, and suffix (if applicable) are required and will appear in PubMed.

*First Name and Middle Initial(s)	*Last Name	*Suffix (eg, Jr, III)	Academic Degrees	Institution	Location (city, state/province, country)	Role or Contribution, eg, chair, principal investigator	Group (if more than 1 Group listed in the byline) and/or Subgroup (eg, Steering Committee)
Joanna	Xu		RRT	BC Children's Hospital	Vancouver, British Columbia, Canada	Research Assistant	READAPT-Kids
Zainab	Zeyan		BS	BC Children's Hospital	Vancouver, British Columbia, Canada	Research Assistant	READAPT-Kids
Baneesh	Khosa		BASc (c)	BC Children's Hospital	Vancouver, British Columbia, Canada	Research Assistant	READAPT-Kids
Gordon	Krahn		BSc RRT	BC Children's Hospital	Vancouver, British Columbia, Canada	Research Assistant	READAPT-Kids
Trish	Page		RN MSN	BC Children's Hospital	Vancouver, British Columbia, Canada	Research Assistant	READAPT-Kids
Rita	Janke		RN MSN	BC Children's Hospital	Vancouver, British Columbia, Canada	Research Assistant	READAPT-Kids
