# Supplementary material for: Risk Factors for Severe Disease Among Children Hospitalized With Respiratory Syncytial Virus
Source: JAMA Netw Open. 2025 Apr 11;8(4):e254666. doi: 10.1001/jamanetworkopen.2025.4666 (PMC11992603; doi:10.1001/jamanetworkopen.2025.4666)
Supplement: Supplement 3. — Data Sharing Statement [file jamanetwopen-e254666-s003.pdf]

Data Sharing Statement

Kirolos. Risk Factors for Severe Disease Among Children Hospitalized With Respiratory Syncytial Virus. *JAMA Netw Open*. Published April 11, 2025.
doi:10.1001/jamanetworkopen.2025.4666

Data
Data available: Yes
Data types: Deidentified participant data
How to access data: Contact Peter Gill (peter.gill@sickkids.ca) for requests to share data.
When available: With publication

Supporting Documents
Document types: None

Additional Information
Who can access the data: Researchers whose proposed use of the data has been approved and aligns with the objectives of the study.
Types of analyses: For any purpose
Mechanisms of data availability: With investigator support after approval of proposal by study team.
